# The Metastatic Bone Marrow Niche in Neuroblastoma: Altered Phenotype and Function of Mesenchymal Stromal Cells

**DOI:** 10.3390/cancers12113231

**Published:** 2020-11-02

**Authors:** Caroline Hochheuser, Lieke M. J. van Zogchel, Marion Kleijer, Carlijn Kuijk, Simon Tol, C. Ellen van der Schoot, Carlijn Voermans, Godelieve A. M. Tytgat, Ilse Timmerman

**Affiliations:** 1 Sanquin Research and Landsteiner Laboratory, Department of Hematopoiesis, Amsterdam UMC, University of Amsterdam, 1066 CX Amsterdam, The Netherlands; c.h.hochheuser@prinsesmaximacentrum.nl (C.H.); m.kleijer@sanquin.nl (M.K.); c.kuijk@sanquin.nl (C.K.); c.voermans@sanquin.nl (C.V.); g.a.m.tytgat@prinsesmaximacentrum.nl (G.A.M.T.); 2 Department of Pediatric Oncology, Princess Maxima Center for Pediatric Oncology, 3584 CS Utrecht, The Netherlands; L.M.J.vanZogchel@prinsesmaximacentrum.nl; 3 Sanquin Research and Landsteiner Laboratory, Department of Experimental Immunohematology, Amsterdam UMC, University of Amsterdam, 1066 CX Amsterdam, The Netherlands; e.vanderschoot@sanquin.nl; 4 Sanquin Research and Landsteiner Laboratory, Department of Molecular and Cellular Hemostasis, Amsterdam UMC, University of Amsterdam, 1066 CX Amsterdam, The Netherlands; s.tol@sanquin.nl

**Keywords:** neuroblastoma, mesenchymal stromal cell, bone marrow, microenvironment, metastasis

## Abstract

**Simple Summary:**

Patients with metastatic neuroblastoma are at risk for relapse after conventional therapy. It is believed that residual neuroblastoma cells in the bone marrow (BM) are responsible for this process after becoming resistant to chemotherapy. Studies in various tumor types suggest that cells in the BM environment (specifically the so-called mesenchymal stromal cells (MSCs)) can promote survival and chemoresistance of tumor cells. We characterized the MSC-compartment within the BM of patients with neuroblastoma, with and without metastases. Our data indicate that the quantity and functionality of the MSC population is altered in the presence of tumor cells. We furthermore identify a specific MSC-subpopulation which is exclusively detected in the presence of tumor cells. These data contribute to the understanding of the interactions between neuroblastoma cells and the environment, which is essential in order to target the tumor cells in the BM more effectively, thereby improving therapy success and preventing relapse.

**Abstract:**

*Background*: The bone marrow (BM) is the main site of metastases and relapse in patients with neuroblastoma (NB). BM-residing mesenchymal stromal cells (MSCs) were shown to promote tumor cell survival and chemoresistance. Here we characterize the MSC compartment of the metastatic NB BM niche. *Methods*: Fresh BM of 62 NB patients (all stages), and control fetal and adult BM were studied by flow cytometry using well-established MSC-markers (CD34−, CD45−, CD90+, CD105+), and CD146 and CD271 subtype-markers. FACS-sorted BM MSCs and tumor cells were validated by qPCR. Moreover, isolated MSCs were tested for multilineage differentiation and Colony-forming-unit-fibroblasts (CFU-Fs) capacity. *Results*: Metastatic BM contains a higher number of MSCs (*p* < 0.05) with increased differentiation capacity towards the osteoblast lineage. Diagnostic BM contains a MSC-subtype (CD146+CD271−), only detected in BM of patients with metastatic-NB, determined by flow cytometry. FACS-sorting clearly discriminated MSC(-subtypes) and NB fractions, validated by mRNA and DNA qPCR. Overall, the CD146+CD271− subtype decreased during therapy and was detected again in the majority of patients at relapse. *Conclusions*: We demonstrate that the neuroblastoma BM-MSC compartment is different in quantity and functionality and contains a metastatic-niche-specific MSC-subtype. Ultimately, the MSCs contribution to tumor progression could provide targets with potential for eradicating resistant metastatic disease.

## 1. Introduction

Neuroblastoma (NB) is a pediatric malignancy that accounts for 15% of all cancer-related deaths in children [1]. NB commonly develops in the adrenal gland, but rapidly spreads to other areas of the body, preferentially bone marrow (BM). More than 90% of patients with high-risk disease present with BM metastases at diagnosis [2]. The level of BM infiltration at diagnosis correlates with outcome [3,4]. High risk NB therapy consists of a multimodality approach, including chemotherapy, autologous stem cell transplantation (ASCT) and immunotherapy [5]. Despite this intensive treatment, relapse frequently occurs and long-term survival of patients with metastatic-NB is still less than 40%. BM is the most common site of recurrence; and therapy resistance of tumor cells in BM remains a major obstacle to the cure of NB [6].

The BM microenvironment, also named the ‘metastatic niche’, is considered a ‘fertile soil’ for tumor cells and supports their survival and growth [7]. The BM microenvironment consists of multiple cell types, collectively termed the stromal compartment: mesenchymal stromal cells (MSCs), which support hematopoiesis and contribute to bone homeostasis [8,9], and progeny cells derived from MSCs, such as osteoblasts, adipocytes and chondrocytes. Emerging data suggests that BM niche alterations play a role in the pathogenesis of several adult hematological malignancies and BM-metastatic diseases [7,10]. In breast cancer, for example, metastasized tumor cells have been shown to invade and hijack the function of the BM niche [11,12]. By modifying the niche, tumor cells create an even more hospitable environment for further tumor colonization and expansion within the BM. Modified MSCs can contribute to tumor progression by increasing proliferation and metastatic potential and promoting chemoresistance of cancer cells, making them potential therapeutic targets [13,14,15,16]. For NB, the BM niche is a relatively unexplored field, but based on findings from other cancer types, it’s a promising avenue to explore for drug discovery [17].

In NB, various studies indicated a tumor-supportive effect of MSCs in regards to NB proliferation, survival [18,19,20,21] and chemoresistance [22,23,24]. Although the underlying mechanisms are not fully understood, co-culture studies with NB cell lines suggest that extracellular vesicles and Interleukin-6 (IL-6) play a role. Additionally, some studies with adult cancers suggest that MSCs might induce epithelial to mesenchymal transition (EMT) [25,26,27]. In order to shed light on a potential role of MSCs in the metastatic tumor microenvironment of NB, we analyze primary patient material and characterize the MSC compartment in regards to composition and functionality ex vivo. 

## 2. Results

### 2.1. The Metastatic BM-Niche Contains a Higher Number of MSC

To study how the presence of metastatic NB-cells affects the composition of the BM niche, we studied diagnostic BM aspirates from patients with (*n* = 34) and without BM-metastases (*n* = 15). Aspiration volume and BM-MNC concentration did not differ significantly between localized and metastasized patients (Figure S1A,B). As controls, we analyzed BM samples of patients with other (non-NB) tumors, comprising ganglioneuroma (GN), ganglioneuroblastoma (GNB), and malignant peripheral nerve sheath tumor (MPNST) (relevant patient information is summarized in Table S1). The extent of BM infiltration was determined by a highly sensitive and specific NB-marker panel as previously developed [28,29], consisting of PHOX2B, TH, CHRNA3 and GAP43. BM-infiltration was classified as [<0.01%], [<0.1%], [0.1–10%], or [>10%], according to an external standard. In 50% of the BM samples of patients that have been diagnosed with localized disease—that is, no evidence of metastases when assessed by ^123^I-MIBG scintigraphy, morphological examination of BM smears and immunohistochemistry of trephine biopsies—tumor cells were detected by RT-qPCR. In all subsequent analyses, we define metastatic disease in the BM based on qPCR positivity as BM^pos^ and qPCR negativity as BM^neg^.

**Figure 1:**
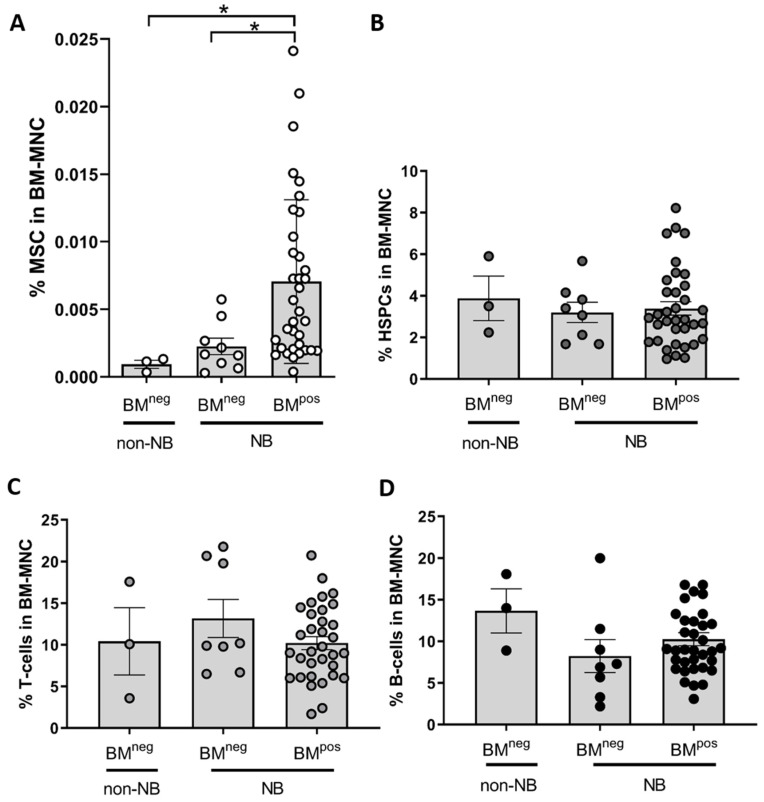
The MSC-frequency is significantly increased in bone marrow with versus without metastases. The frequency of 4 different BM cell populations relative to the number of BM mononuclear cells (BM-MNC) was analyzed by flow cytometry in diagnostic BM aspirates of patients with neuroblastoma and other tumors. Metastatic disease in the BM is defined based on qPCR positivity as BM^pos^ and qPCR negativity as BM^neg^. Non-NB tumors analyzed are localized ganglioneuroma (GN; *n* = 1), ganglioneuroblastoma (GNB; *n* = 1) and malignant peripheral nerve sheath tumor (MPNST; *n* = 1). (**A**) CD34−, CD45−CD90+CD105+ mesenchymal stromal cells (MSCs) (BM^pos^ *n* = 35, BM^neg^ *n* = 9) (**B**) CD34-positive CD45-dim hematopoietic stem and progenitor cells (HSPCs) (**C**) CD3-positive T-cells and (**D**) CD19-positive B-cells. For B-D: BM^pos^ *n* = 35, BM^neg^ *n* = 8. Only statistically significant results as determined by Kruskal-Wallis test are indicated: (*) *p* < 0.05.

First, the number of MSCs were determined by flow cytometry using the classical MSC-marker panel [30] (CD34−, CD45−, CD90+, CD105+). In the NB BM samples, a significantly higher percentage of MSCs was detected in BM^pos^ samples compared to BM^neg^ samples (Figure 1A). We also studied the frequency of other BM-residing cells that have been implicated in tumor context, including hematopoietic stem and progenitor cells (HSPCs), T-cells and B-cells which were detected by expression of cell surface markers CD34/CD45, CD3 and CD19, respectively. No significant differences in frequency for these three cell types were found between BM^pos^ and BM^neg^ samples (Figure 1B–D). Based on the altered frequency of MSCs in the metastatic BM niche, we continued to investigate the MSC compartment in more detail. 

### 2.2. Exclude Detection of Tumor Cells with MSC-FACS-Panel

**Figure 2:**
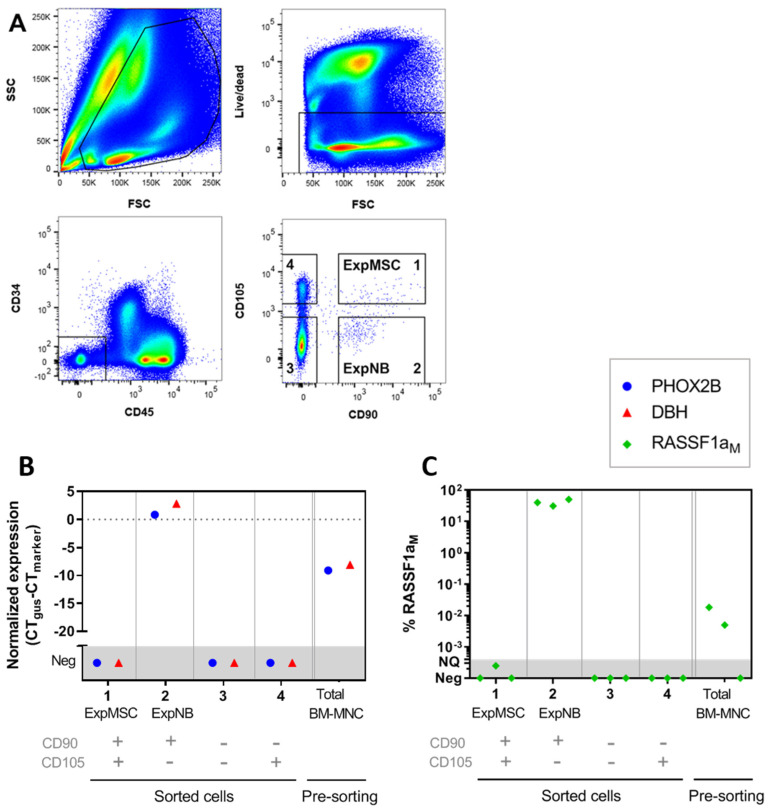
qPCR NB-marker testing in FACS-sorted cell populations proved that we can confidently discriminate between MSC and NB-cells in samples of metastatic-NB patients. (**A**) Representative plots to illustrate the gating strategy for isolating MSCs from bone marrow mononuclear cells (BM-MNCs) of a patient with metastatic-NB. Cells of population #1 express the classical MSC-markers CD90 and CD105, and are therefore expected to all belong to the MSC compartment (ExpMSC). Population #2 (CD90+ CD105−) is expected to contain NB-cells (ExpNB). (**B**,**C**) Testing of NB-marker expression by qPCR (**B**) *PHOX2B* and *DBH*; analyzed in triplicate on BM of a metastatic NB patient; (**C**) methylated *RASSF1a*; analyzed in triplicate on BM of 3 metastatic NB patients. All three NB-markers were detected in sorted NB-cells, but not in the MSC population. For RNA markers (*PHOX2B* and *DBH*): b-glucuronidase (*GUSB*) was used for normalization (normalized Ct (deltaCt) = Ct *GUSB* − Ct marker). For DNA marker *RASSF1aM*: qPCRs were performed specific for its methylated (*RASSF1aM*) and unmethylated (*RASSF1aU*) status. The percentage of RASSF1a methylation was defined as: [*RASSF1aM*]/([*RASSF1aM* + *RASSF1aU*]). qPCR reactions were carried out at least in duplicate and mean values were used. Neg = negative, NQ = not quantifiable.

Since NB cells and MSCs share the expression of certain markers like CD90 and GD2, we performed qPCR testing on FACS-sorted MSCs from BM^pos^ in order to: (i) ascertain that we can confidently discriminate between MSC and NB during FACS isolation and thereby (ii) demonstrate that the increase in MSC frequency is not caused by erroneously including NB cells in the analysis. Figure 2A shows the gating strategy to isolate MSCs from cryopreserved BM. First, viable CD34 and CD45 negative cells were selected. From this CD34− CD45− cell population, we used CD90 and CD105 to further isolate four populations: (1) The CD90+CD105+ MSC population that we expect to be MSCs, hence named ‘Expected-MSC (ExpMSC)’, (2) CD90+CD105− population, containing CD90-expressing tumor cells [31,32], hence named ‘Expected-NB (ExpNB)’, (3) CD90−CD105− population, and (4) CD90−CD105+ population.

We used *PHOX2B* and *DBH* as mRNA markers and *RASSF1a_M_* as an NB-specific DNA marker to identify NB-cells [28,29]. This panel discriminates between NB-cells and MSCs, and also detects both adrenergic (ADRN) NB-cells and mesenchymal (MES) NB-cells [33] (Figure S2A). We did not detect expression of these markers in culture-expanded MSCs or control BM samples of adult donors (Figure S2B,C). *RASSF1a_M_*_,_ *PHOX2B* and *DBH* were detected in the pre-sorting patient BM sample (total BM-MNCs) and in the sorted ‘ExpNB’ population (#2). Importantly, all three NB-markers were not detected in the ‘ExpMSC’ population (#1) (Figure 2B,C), pointing out that the gating strategy reliably distinguishes between MSCs and NB-cells. Therefore, the increase in MSC frequency we observed in BM^pos^ (see Figure 1A) is not attributable to detection of metastatic tumor cells.

### 2.3. MSC in the Metastatic BM-Niche Differ Functionally and Contain a Specific Subtype

MSCs are defined by their clonogenic potential and trilineage differentiation capacity. To study potential functional alterations of MSCs in the metastatic BM-niche, we examined the MSC compartment for these characteristics [30].

**Figure 3:**
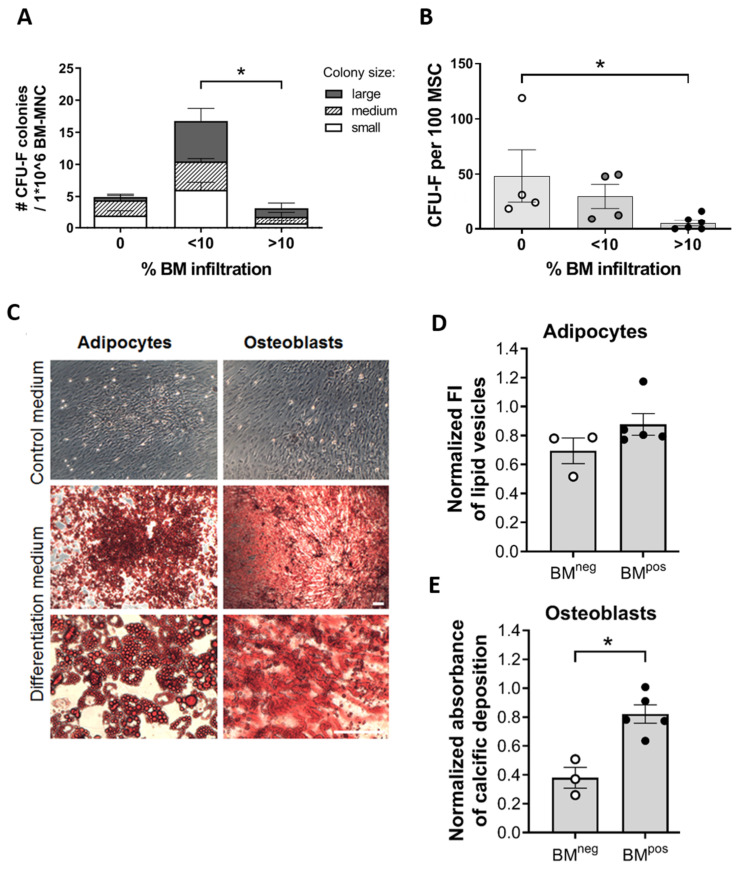
The MSC compartment of metastatic BM is characterized by a lower CFU-F capacity per 100 MSCs and an increased differentiation potential towards osteoblasts. (**A**,**B**) The clonogenic potential of MSCs was determined by a Colony-forming unit-fibroblast (CFU-F) assay to determine the frequency of stromal progenitors. BM infiltration is determined by qPCR. (**A**) Average colony number per 1 × 10^6^ MNCs of BM as well as colony size from NB-patients with and without BM metastases. Experiments were carried out in triplicate. (**B**) number of colonies per 100 MSC for NB-patients with and without BM metastases. (**C**–**E**) Differentiation capacity of MSCs. (**C**) MSCs isolated from BM of localized- and metastatic-NB patients and adult BM, were assayed for their differentiation capacity. After 3 weeks of culturing in the respective differentiation medium, cells were stained for lipid vesicles of adipocytes (using Oil red O) or for calcium deposits, as an indicator of osteogenesis (Alizarin Red S), and nuclei (Hoechst). Scale bar = 50 µm (**D**,**E**) Quantification of differentiated cells. Metastatic disease in the BM is defined based on qPCR positivity as BM^pos^ and qPCR negativity as BM^neg^ (D: adipocytes, E: osteoblasts). (*) *p* < 0.05.

**Figure 4:**
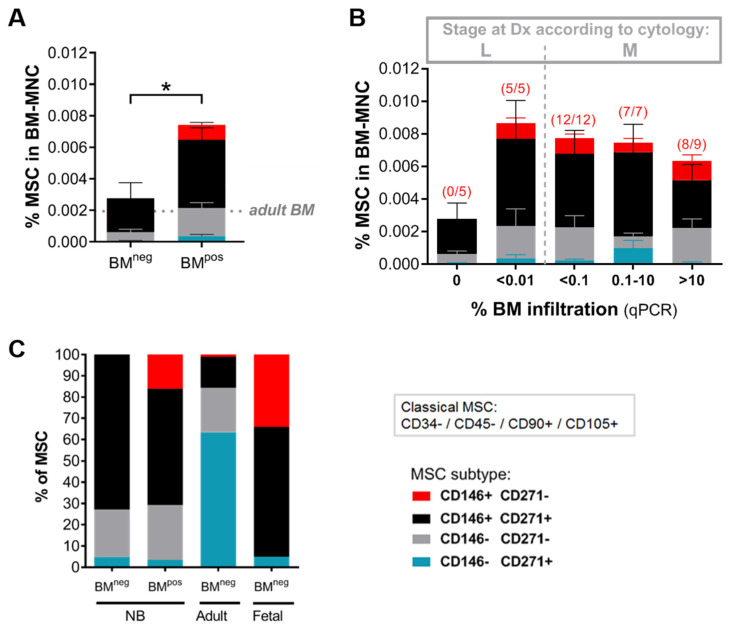
The metastatic BM niche contains a specific MSC subtype. Metastatic disease in the BM is defined based on qPCR positivity as BM^pos^ and qPCR negativity as BM^neg^ (**A**) Frequency of MSCs in diagnostic BM aspirates of patients relative to the number of BM-mononuclear cells (BM-MNC) (BM^pos^ *n* = 33, BM^neg^ *n* = 5). MSCs are detected by flow cytometry using the classical MSC-panel (CD34−, 45−, 90+, 105+) and subtype distribution is analyzed based on CD146 and CD271 expression. Dotted line marks average MSC level in control adult BM (*n* = 5). (*) *p* < 0.05. (**B**) MSC (-subtype) levels in BM samples grouped based on stage and extent of BM infiltration (as determined by qPCR). Patients were diagnosed as ‘localized’ when there was no evidence of metastases by ^123^I-MIBG scintigraphy and morphological examination of BM smears/biopsies. Based on RT-qPCR results, we classified them as negative (*n* = 5) or positive. qPCR positive localized BM samples (*n* = 5) all contain less than 0.01% tumor cells. For stage 4 NB-patients (i.e., with metastases) the extent of BM infiltration is classified as <0.1% (*n* = 12), 0.1–10% (*n* = 7), or >10% (*n* = 9). Number of BM samples positive for CD146+CD271− MSC is indicated in brackets above the bar. (**C**) Relative contribution of distinct MSC subtypes to the MSC compartment in diagnostic BM of NB-patients compared to fetal and adult BM. Adult MSCs: *n* = 4, fetal MSCs: *n* = 6. Data from fetal MSCs is adapted from Maijenburg et al., 2012 [36].

Their clonogenic potential was assessed in colony forming unit–fibroblast (CFU-F) assays. In BM with <10% NB infiltration we detected more and bigger colonies than in the group without metastases. This effect might arise because the total number of MSCs is increased in metastatic BM (see also Figure 1A and Figure 4B). In the ‘BM > 10%’ group the absolute number of colonies formed was significantly lower than in the <10% group (Figure 3A) which points towards a reduced clonogenic capacity of MSCs in BM with higher NB infiltration. Indeed, when calculating the number of colonies per 100 MSCs as an indication of the MSC clonogenic potential, we observed a decrease in clonogenicity with increasing BM-infiltration (Figure 3B). The proliferative capacity, assessed as population doubling time (PDT), demonstrated no difference for MSC from BM^pos^ and BM^neg^ samples (Figure S3A) indicating that the proliferation of the total MSC population does not only depend on the progenitor cells with clonogenic potential. 

Next we studied one of the MSCs defining characteristics: the differentiation capacity. After isolation from BM, MSCs were cultured in specific medium to direct their differentiation toward the osteogenic and adipogenic lineages (Figure 3C). While the adipogenic differentiation capacity did not vary between MSCs from BM^pos^ and BM^neg^ (Figure 3D), MSCs from BM^pos^ were more prone to differentiate towards osteoblasts compared to MSCs from BM^neg^ (Figure 3E). The data emphasize that MSCs from BM^pos^ not only differ in quantity, but also display functional alterations.

We further added CD271 (low-affinity nerve growth factor receptor, LNGFR) and CD146 (melanoma cell adhesion molecule, MCAM) to the FACS panel to further characterize MSC subtypes (gating strategy in Figure S4A) [34,35].

In BM^pos^ we identified a CD146+CD271- MSC subtype (hereinafter referred to as ‘subtype’) which was not detectable in BM without NB metastases (Figure 4A). Furthermore, all of the samples (*n* = 5) of patients that had supposedly no BM-invasion but tested qPCR positive for our NB-marker panel (as described above), contained the subtype (Figure 4B). 

Of note, this MSC subtype constitutes 18.2% of the ‘ExpMSC’ population in a representative sample (Figure S4B) which tested negative by qPCR for NB markers (see Figure 2B,C). MSC- and subtype- levels did not significantly correlate with the extent of BM infiltration (Figure 4B). These data demonstrate an association between the presence of tumor cells and the MSC subtype in primary patient material. Flow cytometry revealed a high-GD2 expression in these CD146+CD271− cells compared to total MSCs (Figure S4C). The percentage of subtype cells within the MSC compartment did not differ significantly between patients with or without MYCN amplification/gain (Figure S4D). Interestingly, this MSC subtype was also detected in healthy fetal BM [36], while in adult BM it constituted merely ~1% (Figure 4C). Furthermore, we have analyzed the subtype marker expression in cultured MSCs and have found that compared to primary MSCs from BM samples, the cultured MSCs gain expression of CD146 and lose expression of CD271 (Ilse Timmerman, Carlijn Voermans. Sanquin Research and Landsteiner Laboratory, Department of Hematopoiesis, Amsterdam UMC, University of Amsterdam, Amsterdam, Netherlands. Personal observation, 2018).

### 2.4. The MSC Compartment in the Course of Treatment

After having identified alterations in the diagnostic MSC compartment of metastatic BM samples, we studied whether and how the high-risk treatment influences the total MSC frequency as well as the presence of the CD146+CD271− subtype within the BM-MSC population. 

**Figure 5:**
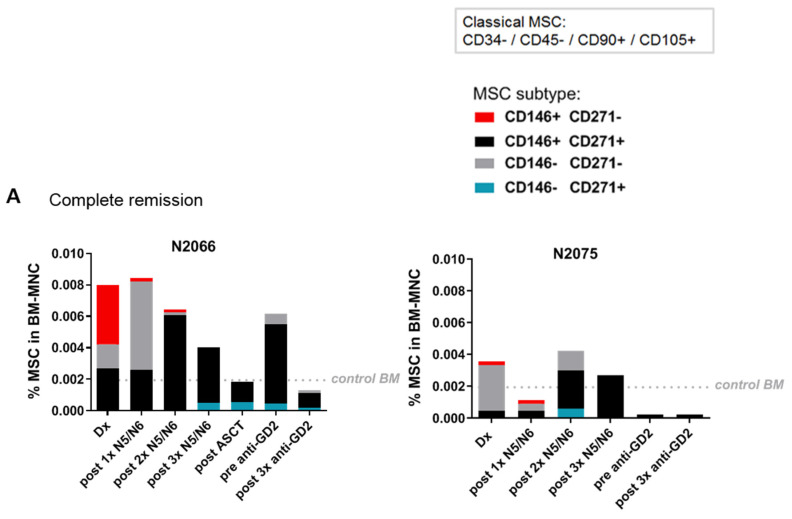

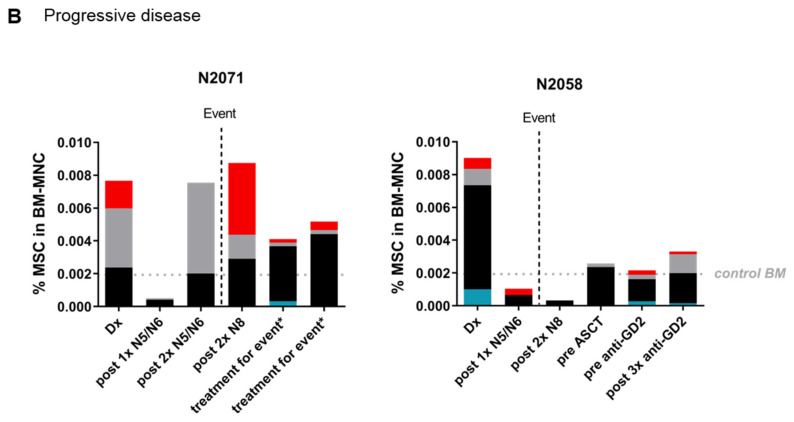
The MSC subtype disappears during treatment and is detected again at relapse. MSC (-subtype) levels in follow-up BM samples of representative high-risk patients with metastatic disease as determined by flow cytometry (**A**) The subtype disappears after treatment in BM of patients in complete remission (**B**) In patients with an event the subtype is detected again after the event or at relapse. If progressive or refractory disease was observed during treatment, induction therapy was extended with additional courses of N8 [37]. * Due to refractory disease after additional courses of N8, patient 2071 was included in the BEACON trial [38]. Dx: diagnosis. ASCT: autologous stem cell therapy. N5/N6: The standard treatment regimen can be found in Figure S5.

The treatment of high-risk NB consists of a multi-modal approach, including several courses of induction chemotherapy (N5/N6) with subsequent consolidation therapy consisting of autologous stem cell transplantation (ASCT) and anti-GD2 immunotherapy as post-consolidation (Figure S5). We tested follow-up samples of our patient cohort during treatment and demonstrate the presence of the subtype in four representative patient cases (Figure 5). The CD146+CD271− -subtype disappeared completely during induction treatment in BM of two patients in complete remission (Figure 5A). In two patients with an event, on the other hand, the MSC-subtype disappears during chemo treatment but is detected again at relapse (Figure 5B). Furthermore, we observe that the overall MSC level is declining from diagnosis until end of treatment/relapse in all four patients.

**Figure 6:**
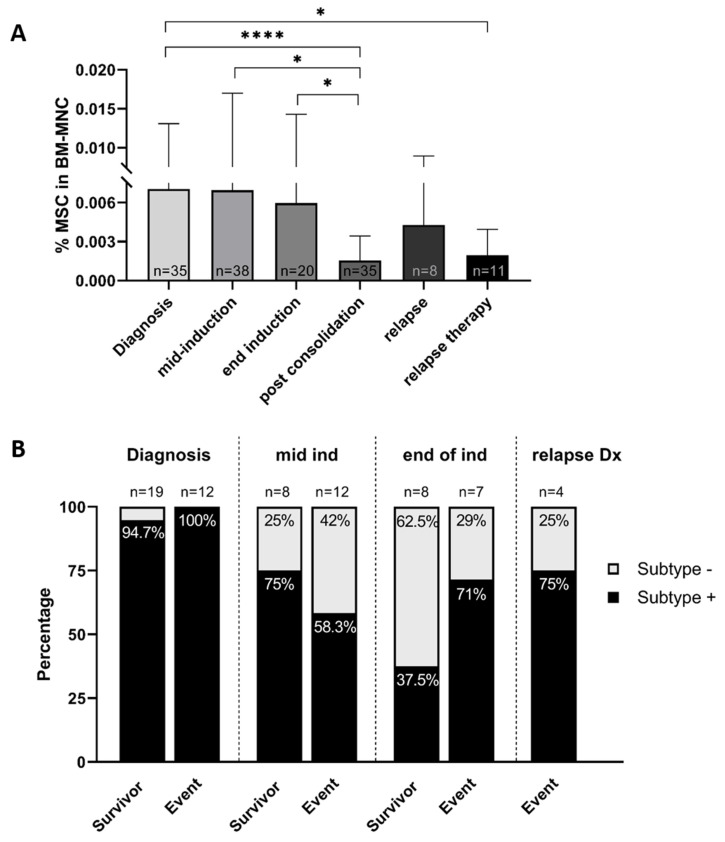
(**A**) Flow cytometry analyses on BM aspirates of patients with metastatic disease at diagnosis and throughout treatment. MSC frequency relative to the number of BM mononuclear cells (BM-MNC) is analyzed and displayed as the average of all samples in the respective treatment group. Sample size per group is indicated at the bottom of each bar. * *p* < 0.05, **** *p* < 0.0001. (**B**) Distribution of subtype positive and -negative samples at diagnosis, mid induction, end of induction and at relapse, for patients who had an event and patients who remained in complete remission (survivor). Sample size is indicated above each bar and percentages of subtype positive/negative samples within the group are indicated within the bars. Mid induction includes all samples after 2–5 courses of induction chemotherapy, unless additional courses were given after 6 courses (in that case samples before the last course were also included in the mid-induction timepoint).

In order to consolidate the latter finding we analyzed the MSC frequency in the BM across different treatment stages in 50 patients (35 diagnostic and 112 follow-up samples). We found that the MSC levels are indeed significantly reduced after long exposure to chemo- and immune therapy: At diagnosis and mid-induction the quantity of MSCs is the highest and decreases to its lowest value during post-consolidation and relapse therapy (Figure 6A). The total number of BM-MNCs in these samples does not differ significantly (Figure S6A). 

After detecting a difference in subtype occurrence between patients in complete remission and with progressive disease (see Figure 5), we investigated the association between clinical outcome and the presence of the CD146+CD271− subtype in additional follow-up samples of our cohort. For this analysis we included 70 samples: 31 at diagnosis, 20 mid induction, 15 at the end of induction and four at relapse. While at diagnosis 30 of 31 BM samples contained the subtype regardless of the clinical outcome, we see that after induction therapy (“end ind”) the subtype predominantly remains present in those patients that have an event (i.e., relapse, progressive disease or refractory disease) (71%). In patients who remain in complete remission, this subset is only detected in 37.5%. In samples from patients that suffer a relapse we detect the subtype in 75% of the samples (Figure 6B). We sought to establish whether after induction therapy the presence of the subtype statistically correlates with the clinical outcome of these patients. While the subtype seems more prevalent in samples from the patients that have an event, this was not significant due to the small sample size of this cohort (*p* = 0.31). We furthermore analyzed these samples as well as 15 additional samples from the post-consolidation and treatment after relapse group for concordance of subtype- and NB cell presence by qPCR. In 75% of the samples the presence of the subtype was indeed concordant with qPCR positivity for the NB-qPCR-panel. However, discrepancies were found with both subtype^+^/qPCR^−^ (*n* = 11) and subtype^−^/qPCR^+^ samples (*n* = 10) (Figure S6B). 

## 3. Discussion

Considering the high frequency of recurrent NB-tumors in BM, there is an urgent need to establish a better understanding of this metastatic niche. In some cancer types, a tumor-supportive effect of MSC in the BM niche has been established [12,14]. Whether the same mechanisms are being employed by NB to modify the microenvironment has not been proven, but evidence from in vitro studies indeed points towards tumor supportive effects of MSC [18,19,20,21]. Using fresh patient material from diagnostic BM samples, we show here that the BM microenvironment is altered when NB-cells are present. As controls, we analyzed BM-samples of other (non-NB) malignancies or adult (and fetal) BM since BM samples of healthy, age-matched individuals, as an ideal control group, are hardly available due to ethical restraints. This limitation needs to be considered in the interpretation of results since a natural difference between fetal, pediatric and adult BM exists [36]. 

First, we demonstrated an increased MSC frequency in the presence of NB cells. At this point we cannot conclude whether this might be a mechanism of tumor defense by the body (if MSCs act tumor-suppressive) or whether NB cells have hijacked the MSC and actively induce MSC proliferation. Based on evidence from literature implicating a tumor-supportive role of MSCs, an increased frequency of MSCs might point towards a positive feedback loop with NB cells in vivo: An increased number of MSCs would entail even stronger promotion of NB proliferation and vice versa.

Next to MSCs, we analyzed hematopoietic stem and progenitor cells (HSPCs), T-cells and B-cells. The number of HSPCs is relevant in the context of the metastatic BM niche since the presence of tumor cells has been described to alter their cell number and functionality [39]. The quantity and quality of the HSPC compartment is also of importance for the harvest of HSPCs for autologous stem cell transplantation (ASCT) [40], a crucial part in the treatment of NB. The relative frequency of T- and B-cells in the metastatic BM can be altered in presence of tumor cells due to the immunosuppressive functions of the metastases [41]. The frequency of all three cell types, however, did not differ between BM^pos^ and BM^neg^ samples. Therefore, the stromal compartment in the BM seemed to be a candidate worth investigating. 

We found that MSCs from BM^pos^ not only differ in quantity but also functionally. The clonogenic potential was reduced with increasing extent of BM infiltration, an effect that has similarly been described for multiple myeloma (MM)- and acute myeloid leukemia (AML)-associated MSCs [42,43]. Previous work of our group has shown that the CD271+ fraction of BM-MSCs holds the most clonogenic potential [36], and the fraction of these cells is reduced in BM^pos^ compared to BM^neg^ or adult BM (see Figure 4C). It is conceivable that with increasing tumor infiltration, the MSCs’ purpose is more diverted towards tumor-related functions than to maintaining their self-renewal capacity. The remaining MSCs in BM^pos^ that do retain their clonogenicity function tend to form larger colonies, a feature typical for more primitive progenitors [44].

In line with an overall decreased clonogenic potential is the finding that MSCs from BM^pos^ display a pronounced tendency to differentiate into osteoblasts in the presence of NB cells. This suggests an involvement of MSCs in the dysregulated bone homeostasis within the metastatic BM environment, which has been described for various tumor types that metastasize to the BM [45]. While the differentiation potential of MSCs did not differ in the context of acute lymphoblastic leukemia (ALL) [46], insights from studies in AML, prostate cancer and head and neck squamous cell carcinoma (HNSCC) showed that tumor cells do induce a pre-osteoblast-rich niche in the BM or that they prime MSCs towards the osteogenic lineage [47,48,49]. Importantly, an in vitro study with NB cell lines demonstrated that NB cells enhance BMP-4-driven osteoblast differentiation of BM-MSCs by increasing MSC-internal VEGFA expression [50]. What the advantages of this increased osteoblastic differentiation are, is not known in the context of NB. Studies in prostate cancer, however, showed that osteoblast-derived signaling can directly increase tumor cell proliferation [51]. Contrary to these findings, BM metastases of NB have also been described to involve mainly osteolytic processes [50]. These seemingly contradictive evidences (osteoblastic vs. osteolytic) are in fact not surprising since bone formation and –destruction represent “two extremes of a continuum” [45] and might thus be coinciding events that have to be kept in balance for a functional microenvironment. The details of MSCs’ contribution to the intricate regulation of bone homeostasis in NB remain to be elucidated.

A further striking difference in the MSC compartment between BM^pos^ and BM^neg^ is the presence of a CD146+CD271− subtype. In the healthy adult BM environment bone-lining MSCs have been demonstrated to express CD271 alone, whereas CD146-expressing MSC located in perivascular regions [35]. CD146 has furthermore been described to define a bone-forming subpopulation of MSCs [52], which is interesting considering our finding that NB-MSCs have an increased osteogenic differentiation capacity. In our analysis we found that the subtype correlates strongly with tumor cell presence. The question might arise whether these CD146+CD271− cells are truly MSCs or whether they might constitute a NB cell subtype that gained mesenchymal traits. In the current study, we show that these CD146+CD271− cells are different from NB cells: Firstly, the MSC- and subtype- levels did not significantly correlate with the extent of BM infiltration at diagnosis (Figure 4B). Secondly, the “ExpMSC” population (Figure 2A), of which the subtype constitutes 18.2% in a representative sample (Figure S4B), was negative for the highly sensitive RT-qPCR markers for both ADRN and MES NB cells (Figure 2B,C). Interestingly, our group previously detected this subtype in healthy fetal BM (35.5% of all MSCs), suggesting that it might represent an immature MSC population [36]. Moreover, functional tests on CD146+CD271- cells isolated from fetal BM demonstrated their CFU-F and trilineage differentiation capacity [36], both characteristics of MSCs.

The concrete functions of the MSC subtype in the metastatic niche remain to be elucidated in future studies. The expression of CD146 on these cells might indicate a tumor-associated phenotype since CD146 has previously been implicated to play a role in tumor context: In melanoma, CD146+ perivascular MSCs interacted with tumor cells via CD146 for efficient metastasis [53]. In an in vitro study, CD146 expression on MSCs increased upon exposure to tumor-conditioned medium from glioblastoma and melanoma cell lines [54]. Furthermore, CD146 has been confirmed to be expressed by tumor cells and the fact that its expression correlates with aggressiveness, poor prognosis and metastatic potential [55] implies that it might have a (passive or active) role in tumor progression.

Based on the hypothesis that the subtype is induced by tumor cell presence we next determined the subtype occurrence during the treatment course for individual patients. In two exemplary patients in complete remission the subtype disappears during induction treatment and remains undetected while in 2 patients with progressive disease the subtype also disappears during induction treatment but is detected again at later treatment stages or relapse (Figure 5A,B). These observations support the hypothesis that the subtype might be induced by the tumor cells. 

When analyzing the correlation between subtype-and NB presence in depth, we found that in the majority of cases (75%), the subtype presence was indeed concordant with NB cell presence (as determined by the (ADRN) NB-qPCR-panel). The remaining samples, however, either contained the subtype while being qPCR-negative, or vice versa, tested positive in the qPCR while no subtype was detected (as depicted in Figure S6B). Subtype^+^/qPCR^−^ samples (*n* = 11) suggest that either the MSC subtype might persist while NB cells have already been eradicated by treatment, or that some NB cells remain undetected by the current qPCR panel, since it does not identify mesenchymal tumor cells. This argument could be especially relevant for samples of later treatment stages since mesenchymal tumor cells have been described to be relatively chemoresistant and more prevalent in patients with poor outcome [27,33,56]. On the other hand, in 10 of 85 samples we did not find the subtype, but they were positive by qPCR. These data regarding the discrepant findings must be interpreted with caution because the limited amount of bone marrow material from frozen remains, in combination with the technical limitations to detect the low number of MSCs, could lead to false-negative results in the detection of the subtype. In addition, the low frequency of MSCs can be aggravated through treatment (see Figure 6A). Equally important, poor sample quality in form of blood dilution [57] may result in false-negative qPCR and/or MSC subtype presence and thus contribute to discrepant findings.

Ultimately, we asked whether the presence of subtype cells was associated with clinical outcome. We were not surprised to find no association between presence of the subtype during induction therapy and clinical outcome, given the heterogeneity of the samples that were combined in this treatment group. Instead, we tested whether the presence of subtype cells at the prognostically relevant time point of end of induction therapy correlates with the clinical outcome of the patient. However, some limitations of this study precluded analysis for association with clinical outcome. Firstly, the overall number of samples was too low to compensate for the various technical and biological limitations regarding MSC number in BM samples. The aspiration volume and cellularity of metastatic BM samples varies greatly (Figure S1A,B). Analysis of samples with overall higher cellularity and therefore higher MSC number might therefore allow detection of the subtype in samples of patients with an event which are now categorized as subtype-negative. Furthermore, it is known that some neuroblastoma patients still have minimal residual disease in the BM during or after consolidation, and have complete BM clearance only after post consolidation therapy [58]. Therefore, the end of induction time point might be too early to correlate the subtype presence with clinical outcome of the patient. In the current study, it was not possible to analyze post-consolidation samples instead, which might hold potential for more conclusive results: Several samples had to be excluded from the analysis for the subtype due to insufficient MSC frequency in the BM, especially from later treatment groups (post consolidation, relapse treatment). Due to the resulting low sample size in these two treatment groups as well as a lack of standardized timepoints we had excluded the post consolidation and relapse treatment group from the above described analysis. Future studies with prospectively collected samples at standardized time points and with sample volumes that allow detection of sufficient MSC numbers for subtype analysis will provide more conclusive results to confirm or reject the hypothesis of a prognostic value of the MSC subtype.

Lastly, we had discovered that the number of total MSCs in 147 samples of 50 patients decreased during treatment. While the stromal compartment has been described to be affected by autologous BM transplantation and high-dose radiation already a few decades ago [59,60], the effects of chemotherapy on MSCs in terms of proliferation and functionality have only been established rather recently [61,62]. Our finding that MSC frequency decreases after intense chemotherapy and subsequent ASCT and immunotherapy corroborates the recent findings from in vitro studies.

## 4. Materials and Methods

### 4.1. Patient Samples and Treatment Regime

We analyzed 49 diagnostic and 112 follow up clinical samples from 62 patients: 47 with metastatic and 15 with localized disease. 34/49 diagnostic samples and all follow up samples were derived from patients with metastatic disease. 

Patients were treated in accordance with the Dutch NBL2009 trial (Figure S5), at the Princess Máxima Center for Pediatric Oncology. We used remains of a study approved by METC of the Amsterdam UMC (MEC:07/219#08.17.0836). Events are defined as recurrent disease, progressive and refractory disease. The median follow-up time is 33.1 months. Further patient information can be found in Table S1.

As non-NB controls we included human adult bone marrow mesenchymal stromal cells (ABMSCs) and fetal bone marrow mesenchymal stromal cells (FBMSC) as well as 3 BM samples of patients without BM invasion: one malignant peripheral nerve sheath tumor (MPNST), one ganglioneuroma (GN) and one localized ganglioneuroblastoma (GNB) (with <1% NB cells present in the tumor).

### 4.2. Adult and Fetal Bone Marrow MSCs

ABMSCs and FBMSCs were isolated and cultured as described previously [63]. In summary, ABMSC) were isolated from BM from the sternum of adult patients undergoing cardiac surgery. Fetal bones were flushed with Iscove’s modified Dulbecco’s medium (IMDM) containing 10% FCS and 1% penicillin-streptomycin. MSCs were isolated by density gradient centrifugation (Ficoll-paque, 1.077 g/mL; Cat# 17144003, GE Healthcare, Uppsala, Sweden). The remaining erythrocytes in the cell suspension were lysed using NH4Cl for 10 min on ice. Subsequently, cells were rinsed in PBS. Collection of fetal tissues for research purposes was approved by the medical ethical review board of the Academic Medical Centre (AMC) (MEC: 03/038). The fetal tissue samples were obtained from the HIS facility of the AMC, Amsterdam. All material has been collected from donors from whom a written informed consent for the use of the material for research purposes had been obtained by the Bloemenhove clinic (Heemstede, The Netherlands). These informed consents are kept together with the medical record of the donor by the clinic. Collection of adult bone marrow aspirates for research purposes, after informed consent, was approved by the medical ethical review board of the AMC (MEC:04/042#04 17,370). Donor age in this study ranges from 63 to 74 years with an average of 70 years of age and include both males and females. A total of seven ABMSC donors were included in this study.

### 4.3. NB-BM Sample Preparation

Clinical BM samples of NB patients were collected in EDTA tubes and processed within 24 hours. Prior to 2017, 0.5 mL bone marrow (BM) was transferred to PAXgene Blood RNA Tubes (Qiagen, Hilden, Germany) and stored at −20 °C. The remainder of BM mononuclear cells (BM-MNC) were isolated by Ficoll density gradient centrifugation (Ficoll-paque, 1.077 g/mL; Cat# 17144003, GE Healthcare) and remaining erythrocytes were depleted by NH_4_Cl lysis. Since 2017, 5–10 × 10^6^ cells were stored in RNAbee (Campro Scientific, Berlin, Germany) or Trizol (Invitrogen, Carlsbad, CA, USA) at −80 °C and if applicable, the remainder of the BM-MNC were stored in 10% dimethylsulfoxide at −180 °C.

### 4.4. Flow Cytometry

BM-MNCs for phenotyping were immediately analyzed after isolation from fresh BM samples. Cells were stained with antibodies (see Table S2) for 30 min at 4 °C and washed with PBS + 0.5% FCS + 2 mM EDTA. As negative controls, cells were labeled with appropriate monoclonal isotype controls (BD Biosciences, Franklin Lakes, NJ, USA or Sanquin, Amsterdam, The Netherlands). For the sorting experiments, we optimized the protocol for the use of frozen samples. Frozen BM-MNC samples were thawn in medium containing DNase (100 µg/mL) and MgCl2 (5 mM) and were stained with a live/dead marker (LIVE/DEAD™ Fixable Near-IR Stain, MolecularProbes™, Thermo Fisher Scientific, Waltham, MA, USA) in PBS for 30 min at 4 °C. After washing with PBS + 0.5% FCS + 2 mM EDTA the cells were further processed like the fresh samples as described above.

For phenotyping of BM samples (including determination of absolute cell numbers) and sorting, FACS CantoII and AriaII (BD) were used, respectively. Data was analysed using FacsDiva software (BD) and FlowJo (BD). For determination of subtype distribution within the MSC compartment, samples were excluded of which the total MSC number was less than twice as high as the number of subtype groups. This was especially the case for BM aspirates taken after intensive treatment.

### 4.5. RNA, DNA and (RT) qPCR

The PAXgene Blood Kit (QIAGEN), the RNAbee method (Campro Scientific) or TRIzol (Invitrogen) were used to extract RNA from clinical samples, according to manufacturer’s instructions. RNA was isolated from cell lines using TRIzol (Invitrogen) according to the manufacturer’s instructions. For RNA extraction from the culture-expanded MSC, bulk-sorted MSC and NB cells, the RNeasy Micro Kit (Qiagen, Venlo, The Netherlands) was used, according to manufacturer’s instructions. 

cDNA was synthesized and reverse transcriptase (RT) qPCR, with a maximum of 50 cycles, was performed using Viia7 (Applied Biosystems, Foster City, CA, USA) for β-glucuronidase (GUSB), paired-like homeobox 2B (PHOX2B), tyrosine hydroxylase (TH), growth-associated protein 43 (GAP43), and cholinergic receptor nicotinic α-3 (CHRNA3), as described previously [64] for samples from patients that were diagnosed before 2017. For clinical samples from patients that were diagnosed after 2016, cDNA was synthesized from 2–3 µg RNA, using the High-Capacity RNA-to-cDNA™ Kit (Applied Biosystems) according to manufacturer’s instructions, and samples were tested for the same panel, but TH, GAP43 and CHRNA3 were combined in multiplex (van Zogchel, manuscript in preparation). Sequences and the corresponding fluorophores for the probes for the multiplex qPCR are shown in Table S3. Synthesis of the primers and probes was done by Eurogentec. Reactions were carried out in 20 µL (10 µL TaqMan Multiplex Master Mix (Applied Biosystems), 300 nM forward and reverse primer and 200 nM probe and 5 µL cDNA). Initial heating was done for 20 s at 95 °C, followed by 50 cycles of 1s at 95 °C and 20 s at 60 °C. For both singleplex and multiplex, expression was normalized to GUSB expression using the following equation: [normalized threshold cycle (ΔCT) = (CtGUSB − Ctmarker)]. All RT-qPCR reactions were performed in triplicate (except GUSB, which was performed in duplicate), and mean values were used for analysis. Samples were scored for positivity according to previously published thresholds [64] for singleplex, and new established threshold for multiplex (manuscript in preparation). Quantification with mRNA markers was done according to an external standard (NB cell line IMR-32) and relative values were calculated using the equation 2ΔΔCT (ΔCT sample – ΔCT IMR-32) × 100%. The median relative expression of 5 markers was used for the analysis. BM-infiltration was classified as [<0.1%], [0.1–10%], or [>10%].

### 4.6. DNA Extraction and qPCR

DNA was extracted by using the QIAamp DNA Mini Blood Kit (Qiagen). After DNA isolation, bisulfite conversion was performed using the EpiTect Bisulfite Kit (Qiagen) according to the manufacturer’s instructions. Samples were subsequently analyzed for the epigenetic DNA marker hypermethylated RASSF1a (RASSF1aM) and unmethylated RASSF1A (RASSF1aU), as previously described [29]. Experiments were conducted in triplicate. The percentage of RASSF1a methylation was defined as: [RASSF1aM]/([RASSF1aM+ RASSF1aU]). 

### 4.7. Cell Culture

To obtain MSCs for cell culture, BM-MNC isolated from BM samples were plated in Corning CellBIND^®^ flasks (Cat# CLS3290, Sigma Aldrich, St. Louis, MO, USA) at a concentration of 2.5–3.75 × 10⁵ cells/cm^2^ in DMEM (Cat# 21885025, Thermo Fisher), supplemented with 10% FCS and 1% penicillin-streptomycin (pen/strep). They were cultured for 2 days at 37 °C in a 5% CO_2_ humidified atmosphere, after which non-adhering cells were removed. The remaining adherent cells were MSC, as confirmed by flow cytometry based on the ISCT guidelines [30]. For maintenance of the MSC cultures, cells were detached with TrypLE solution (Gibco, Paisley, UK) by the time 80–90% confluence was reached, and seeded in new culture flasks at a concentration of 3.75 × 10⁴ cells/cm^2^, with a maximum of three passages. NB-cell lines SH-SY5Y, IMR-32, GIMEN, SHEP2, 691B and 691T were cultured as previously described [65,66].

### 4.8. Colony-Forming-Unit-Fibroblast (CFU-F) Assay 

For CFU-F assays 2 × 10^6^ and 4 × 10^6^ BM-MNCs were plated and cultured in MesenCult™ MSC Basal Medium (Cat# 05401, StemCell Technologies, Vancouver, BC, Canada) with MesenCult™ supplement (Cat# 05402, StemCell Technologies). After 14 days, colonies were stained with Coomassie Blue and the number and size of colonies was determined for each condition (Imaris software, Oxford Instruments, Abingdon, UK) (see Figure S3B). Each colony arises from a single progenitor cell, named a CFU-F. By determining in vitro the actual number of colonies, the CFU-F assay therefore determines the in vivo clonogenic capacity of the BM-MSC population.

### 4.9. Differentiation Assays

For MSC lineage differentiation towards adipocytes and osteoblasts, StemPro Adipogenesis Differentiation Kit (A1007001, Gibco) and StemPro Osteogenesis Differentiation Kit (A1007201, Gibco) were used. MSCs of passage 1 were seeded at 2 × 10^4^ cells/cm^2^ and after 1 day medium was replaced by differentiation medium which was refreshed every 3–4 days. After 21 days, cells were fixed with 4% or 10% formaldehyde for osteoblast and adipocyte differentiation, respectively. To study adipogenic differentiation, samples were analyzed for their lipid droplets content by means of Oil Red O staining and for osteogenic differentiation calcium deposits were analyzed by means of Alizarin Red S staining. Cells were furthermore stained with Hoechst dye in order to correct for cell number. Total fluorescence intensity of Oil Red O and Alizarin staining was determined using inverted Zeiss Axiovert 200 widefield microscope (Zeiss, Jena, Germany), corrected for cell numbers (nuclear staining) and normalized to control adult-MSC values. Experiments were carried out in duplicate.

### 4.10. Proliferation Assay

As a measure for proliferative capacity, we determined the population doubling time (PDT). 50.000 MSCs (passage 1) per well were seeded in a 6-well plate. Medium was refreshed every 3–4 days and after 7 days cells were trypsinized and counted. PDT was determined as the average from two passages and calculated using formula: G = t log2/(log Nt − log N0); in which N0 represents the initial population size and Nt the later population size (after t = 7 days). Adult MSCs, derived from the same donor, were used as internal control between experiments. Experiments were carried out in duplicate.

### 4.11. Statistical Analysis

Statistical significance was determined by Kruskal-Wallis tests and Dunn’s test for non-normally distributed continuous variables, Fisher’s exact and Mann Whitney test for analysis of categorical variables. All statistical analyses were performed with GraphPad Prism 8 (GraphPad Software, La Jolla, CA, USA) software. Results were considered significant if *p* ≤ 0.05.

## 5. Conclusions

In conclusion, we have shown in primary patient samples that the MSC compartment is altered in the metastatic niche. Firstly, we demonstrated that we can distinguish between NB cells and MSCs by flow cytometry and were thereby able to detect an increase in total number of MSCs in the presence of NB cells. Secondly, our data indicates a pronounced differentiation capacity of MSCs towards the osteogenic lineage which suggests a role of these cells in the dysregulated bone homeostasis within the metastatic niche. Furthermore, we detect a CD146+CD271− MSC subtype that is exclusively present in metastatic BM samples. Based on insights from studies in other cancer types that implicate CD146 in tumor-MSC interactions, a tumor-related function of the CD146+CD271− cells is conceivable. For future therapy approaches of targeting NB interactions with its microenvironment selectively, it would be of great value to define a tumor-associated MSC subpopulation, so that ‘normal’ MSCs that play a pivotal role in maintaining BM homeostasis could be left intact.

In addition to previously published in vitro studies, this ex vivo data points toward a potential role of MSCs in the metastatic tumor microenvironment. Eventually, the fundamental knowledge about the intricate interactions of NB with MSCs is imperative for finding effective treatment options that target the tumor and its microenvironment in an effective and specific way and thereby avoid unfavorable side effects.

## Supplementary Materials

The following are available online at https://www.mdpi.com/2072-6694/12/11/3231/s1. Figure S1. Aspiration volume and BM-Cellularity measured as concentration MNCs in BM. (**A**) The aspiration volume of BM samples from patients with or without metastases in ml. Differences in cellularity and aspiration volume are not significant as determined by Mann-Whitney test. (**B**) The cellularity of BM samples from patients with or without metastases in cells/mL. Metastatic disease in the BM is defined based on qPCR positivity as BM^pos^ and qPCR negativity as BM^neg^. (BM^pos^ *n* = 10, BM^neg^ *n* = 5). Figure S2: qPCR analysis of NB cell lines, culture-expanded MSCs and control BM samples (**A**,**B**) Selected NB-markers for an optimal RT-qPCR panel to discriminate between NB-cells and MSCs: PHOX2B (blue), DBH (red) and RASSF1aM (green). (**A**) PHOX2B and DBH were expressed in adrenergic (ADRN) NB-cell lines and somewhat lower in mesenchymal (MES) NB-cell lines. RASSF1a was 100% methylated in all ADRN and MES NB-cells. Analyzed in duplicate for each cell line. (**B**) PHOX2B, DBH and RASSF1aM were not detected in culture-expanded MSCs derived from NB-BM and control adult- and fetal-BM. Positive control: ADRN NB cell line IMR32. Analyzed in in 3 donors per group (**C**) Testing of PHOX2B and DBH markers on control BM-MNC (aliquot of BM sample pre-sorting) and CD34-CD45- BM-cell populations, sorted as illustrated in Figure 2. Representative data of an adult control BM; L = stage L, M = stage M, A = adult, F = Fetal, Ctrl = control, + Ctrl = ADRN NB cell line. Figure S3: functional characterization of NB-MSC. (**A**) Population doubling time (PDT) of MSCs in days as a measure for proliferative capacity. PDT is determined for two passages and calculated using formula: G = t log 2/log Nt- log No. Metastatic disease in the BM is defined based on qPCR positivity as BM^pos^ and qPCR negativity as BM^neg^. Experiment was carried out in duplicate. (**B**) Example of quantification and assessment of size of colonies in CFU-F-assay. Imaris software was used to measure number and area of colonies. Upper panel: coomassie blue staining, panel 2: Imaris software was used to set the threshold to determine colony number in region of interest (ROI), panels 3–5: Examples of small, medium and large colony (marked yellow), respectively. Figure S4: Analysis of the CD146+ CD271− MSC subtype (**A**) Representative plot to illustrate the gating strategy for the CD146+CD271− subtype: The CD45− CD34− CD90+ CD105+ fraction (“ExpMSC”, see Figure 2A) was gated further to analyze CD146 and CD271 expression (**B**) Example overview of the sorted cell populations described in Figure 2D, showing the number of sorted cells. Insert: Relative contribution of the CD146+ CD271− subtype to the MSC compartment in BM of metastatic NB-patient (Figure 2D). Testing of expression of CD146 and CD271 showed that 18.2% of the cells of the ExpMSC (#1) population were CD146+ CD271− cells. (**C**) Analysis of GD2 expression on the described CD146+CD271− MSC subtype compared to total MSCs as determined by flow cytometry (**D**) Percentage of CD146+CD271− MSC within the MSC population in diagnostic samples from patients with a tumor without MYCN amplification or gain (MYCN normal), with MYCN gain, or MYCN amplification. There are no differences in the percentage of CD146+CD271− MSCs between the three groups (Kruskal-Wallis test). Figure S5: DGOC NBL2009 treatment protocol. Abbreviations: N5, vindesine, etoposide and cisplatin; N6, vincristine, dacarbacin, ifosfamide, doxurubicin; HD chemo + ASCT, high-dose chemotherapy and autologous stem cell transplantation; EBRT, external beam radiation therapy; IT, anti-GD2 immunotherapy; 13-cis-RA, 13-cis-retinoic acid therapy. Figure S6: (**A**) Absolute number of BM-MNCs in samples that were analyzed for MSC-frequency in Figure 6A (**B**) concordance of FACS- and qPCR analysis of 85 follow-up samples. Absolute number of samples per treatment group, divided into ‘survivor’ and ‘event’ depending on the clinical outcome of the patient. MSCs are detected by flow cytometry using the classical MSC-panel (CD34−CD45−CD90+CD105+) and subtype distribution (subtype+/subtype−) is analyzed based on CD146 and CD271 expression. Presence of tumor cells (NB+/NB−) is determined by qPCR. Table S1: clinical information of NB-patients in this study: Stage according to INSS. Gender: F = female, M = male, MYCNA = MYCN amplification. The median follow-up time is 33.1 months. Table S2: antibodies used for phenotyping and sorting by flow cytometry (as indicated by black marking). Table S3: Primer and probe sequences of the multiplex qPCR.

## References

[1] Park J.R., Eggert A., Caron H. (2010). Neuroblastoma: Biology, prognosis, and treatment. Hematol. Oncol. Clin. N. Am..

[2] Mehes G., Luegmayr A., Ambros I.M., Ladenstein R., Ambros P.F. (2001). Combined automatic immunological and molecular cytogenetic analysis allows exact identification and quantification of tumor cells in the bone marrow. Clin. Cancer Res..

[3] Van Zogchel L.M.J., van Wezel E.M., Stutterheim J., Zappeij-Kannegieter L., van Doornum M., Caron H., Fiocco M., van der Schoot C.E., Tytgat G.A.M. Analysis of the prospective minimal residual disease monitoring study in gpoh-dcog high risk neuroblastoma. Proceedings of the Advances in Neuroblastoma Research Association Meetings.

[4] Viprey V.F., Gregory W.M., Corrias M.V., Tchirkov A., Swerts K., Vicha A., Dallorso S., Brock P., Luksch R., Valteau-Couanet D. (2014). Neuroblastoma mrnas predict outcome in children with stage 4 neuroblastoma: A european hr-nbl1/siopen study. J. Clin. Oncol..

[5] Pinto N.R., Applebaum M.A., Volchenboum S.L., Matthay K.K., London W.B., Ambros P.F., Nakagawara A., Berthold F., Schleiermacher G., Park J.R. (2015). Advances in risk classification and treatment strategies for neuroblastoma. J. Clin. Oncol.

[6] Seeger R.C., Reynolds C.P., Gallego R., Stram D.O., Gerbing R.B., Matthay K.K. (2000). Quantitative tumor cell content of bone marrow and blood as a predictor of outcome in stage iv neuroblastoma: A children’s cancer group study. J. Clin. Oncol..

[7] Shiozawa Y., Eber M.R., Berry J.E., Taichman R.S. (2015). Bone marrow as a metastatic niche for disseminated tumor cells from solid tumors. Bonekey Rep..

[8] Bielby R., Jones E., McGonagle D. (2007). The role of mesenchymal stem cells in maintenance and repair of bone. Injury.

[9] Majumdar M.K., Thiede M.A., Haynesworth S.E., Bruder S.P., Gerson S.L. (2000). Human marrow-derived mesenchymal stem cells (mscs) express hematopoietic cytokines and support long-term hematopoiesis when differentiated toward stromal and osteogenic lineages. J. Hematother. Stem. Cell. Res..

[10] Korn C., Mendez-Ferrer S. (2017). Myeloid malignancies and the microenvironment. Blood.

[11] Price T.T., Burness M.L., Sivan A., Warner M.J., Cheng R., Lee C.H., Olivere L., Comatas K., Magnani J., Kim Lyerly H. (2016). Dormant breast cancer micrometastases reside in specific bone marrow niches that regulate their transit to and from bone. Sci. Transl. Med..

[12] Bliss S.A., Sinha G., Sandiford O.A., Williams L.M., Engelberth D.J., Guiro K., Isenalumhe L.L., Greco S.J., Ayer S., Bryan M. (2016). Mesenchymal stem cell-derived exosomes stimulate cycling quiescence and early breast cancer dormancy in bone marrow. Cancer Res..

[13] Schelker R.C., Iberl S., Muller G., Hart C., Herr W., Grassinger J. (2018). Tgf-beta1 and cxcl12 modulate proliferation and chemotherapy sensitivity of acute myeloid leukemia cells co-cultured with multipotent mesenchymal stromal cells. Hematology.

[14] Baglio S.R., Lagerweij T., Perez-Lanzon M., Ho X.D., Leveille N., Melo S.A., Cleton-Jansen A.M., Jordanova E.S., Roncuzzi L., Greco M. (2017). Blocking tumor-educated msc paracrine activity halts osteosarcoma progression. Clin. Cancer Res..

[15] Karnoub A.E., Dash A.B., Vo A.P., Sullivan A., Brooks M.W., Bell G.W., Richardson A.L., Polyak K., Tubo R., Weinberg R.A. (2007). Mesenchymal stem cells within tumour stroma promote breast cancer metastasis. Nature.

[16] Takam Kamga P., Bassi G., Cassaro A., Midolo M., Di Trapani M., Gatti A., Carusone R., Resci F., Perbellini O., Gottardi M. (2016). Notch signalling drives bone marrow stromal cell-mediated chemoresistance in acute myeloid leukemia. Oncotarget.

[17] Reagan M.R., Rosen C.J. (2016). Navigating the bone marrow niche: Translational insights and cancer-driven dysfunction. Nat. Rev. Rheumatol..

[18] Borriello L., Nakata R., Sheard M.A., Fernandez G.E., Sposto R., Malvar J., Blavier L., Shimada H., Asgharzadeh S., Seeger R.C. (2017). Cancer-associated fibroblasts share characteristics and protumorigenic activity with mesenchymal stromal cells. Cancer Res..

[19] Nakata R., Shimada H., Fernandez G.E., Fanter R., Fabbri M., Malvar J., Zimmermann P., DeClerck Y.A. (2017). Contribution of neuroblastoma-derived exosomes to the production of pro-tumorigenic signals by bone marrow mesenchymal stromal cells. J. Extracell Vesicles.

[20] Silverman A.M., Nakata R., Shimada H., Sposto R., DeClerck Y.A. (2012). A galectin-3-dependent pathway upregulates interleukin-6 in the microenvironment of human neuroblastoma. Cancer Res..

[21] Shankar V., Hori H., Kihira K., Lei Q., Toyoda H., Iwamoto S., Komada Y. (2015). Mesenchymal stromal cell secretome up-regulates 47 kda cxcr4 expression, and induce invasiveness in neuroblastoma cell lines. PLoS ONE.

[22] Lifshitz V., Priceman S.J., Li W., Cherryholmes G., Lee H., Makovski-Silverstein A., Borriello L., DeClerck Y.A., Yu H. (2017). Sphingosine-1-phosphate receptor-1 promotes environment-mediated and acquired chemoresistance. Mol. Cancer.

[23] Wu H.W., Sheard M.A., Malvar J., Fernandez G.E., DeClerck Y.A., Blavier L., Shimada H., Theuer C.P., Sposto R., Seeger R.C. (2019). Anti-cd105 antibody eliminates tumor microenvironment cells and enhances anti-gd2 antibody immunotherapy of neuroblastoma with activated natural killer cells. Clin. Cancer Res..

[24] Ara T., Nakata R., Sheard M.A., Shimada H., Buettner R., Groshen S.G., Ji L., Yu H., Jove R., Seeger R.C. (2013). Critical role of stat3 in il-6-mediated drug resistance in human neuroblastoma. Cancer Res..

[25] Debruyne D.N., Bhatnagar N., Sharma B., Luther W., Moore N.F., Cheung N.K., Gray N.S., George R.E. (2016). Alk inhibitor resistance in alk(f1174l)-driven neuroblastoma is associated with axl activation and induction of emt. Oncogene.

[26] Fischer K.R., Durrans A., Lee S., Sheng J., Li F., Wong S.T., Choi H., El Rayes T., Ryu S., Troeger J. (2015). Epithelial-to-mesenchymal transition is not required for lung metastasis but contributes to chemoresistance. Nature.

[27] Van Groningen T., Koster J., Valentijn L.J., Zwijnenburg D.A., Akogul N., Hasselt N.E., Broekmans M., Haneveld F., Nowakowska N.E., Bras J. (2017). Neuroblastoma is composed of two super-enhancer-associated differentiation states. Nat. Genet..

[28] Stutterheim J., Gerritsen A., Zappeij-Kannegieter L., Kleijn I., Dee R., Hooft L., van Noesel M.M., Bierings M., Berthold F., Versteeg R. (2008). Phox2b is a novel and specific marker for minimal residual disease testing in neuroblastoma. J. Clin. Oncol..

[29] Stutterheim J., Ichou F.A., den Ouden E., Versteeg R., Caron H.N., Tytgat G.A., van der Schoot C.E. (2012). Methylated rassf1a is the first specific DNA marker for minimal residual disease testing in neuroblastoma. Clin. Cancer Res..

[30] Dominici M., Le Blanc K., Mueller I., Slaper-Cortenbach I., Marini F., Krause D., Deans R., Keating A., Prockop D., Horwitz E. (2006). Minimal criteria for defining multipotent mesenchymal stromal cells. The international society for cellular therapy position statement. Cytotherapy.

[31] Bozzi F., Collini P., Aiello A., Barzano E., Gambirasio F., Podda M., Meazza C., Ferrari A., Luksch R. (2008). Flow cytometric phenotype of rhabdomyosarcoma bone marrow metastatic cells and its implication in differential diagnosis with neuroblastoma. Anticancer Res..

[32] Seeger R.C., Danon Y.L., Rayner S.A., Hoover F. (1982). Definition of a thy-1 determinant on human neuroblastoma, glioma, sarcoma, and teratoma cells with a monoclonal antibody. J. Immunol..

[33] Van Wezel E.M., van Zogchel L.M.J., van Wijk J., Timmerman I., Vo N.K., Zappeij-Kannegieter L., de Carolis B., Simon T., van Noesel M.M., Molenaar J.J. (2019). Mesenchymal neuroblastoma cells are undetected by current mrna marker panels: The development of a specific neuroblastoma mesenchymal mrd panel. JCO Precis. Oncol..

[34] Buhring H.J., Treml S., Cerabona F., de Zwart P., Kanz L., Sobiesiak M. (2009). Phenotypic characterization of distinct human bone marrow-derived msc subsets. Ann. N. Y. Acad. Sci..

[35] Tormin A., Li O., Brune J.C., Walsh S., Schutz B., Ehinger M., Ditzel N., Kassem M., Scheding S. (2011). Cd146 expression on primary nonhematopoietic bone marrow stem cells is correlated with in situ localization. Blood.

[36] Maijenburg M.W., Kleijer M., Vermeul K., Mul E.P., Van Alphen F.P., van der Schoot C.E., Voermans C. (2012). The composition of the mesenchymal stromal cell compartment in human bone marrow changes during development and aging. Haematologica.

[37] Berthold F., Faldum A., Ernst A., Boos J., Dilloo D., Eggert A., Fischer M., Fruhwald M., Henze G., Klingebiel T. (2020). Extended induction chemotherapy does not improve the outcome for high-risk neuroblastoma patients: Results of the randomized open-label gpoh trial nb2004-hr. Ann. Oncol..

[38] Activity Study of Bevacizumab with Temozolomide ± Irinotecan for Neuroblastoma in Children. https://ClinicalTrials.gov/show/NCT02308527.

[39] Forest A.E., Shiozawa Y., Pienta K.J., Taichman R.S. (2013). The hematopoietic stem cell niche and bone metastasis. Madame Curie Bioscience Database.

[40] Kraal K., Timmerman I., Kansen H.M., van den Bos C., Zsiros J., van den Berg H., Somers S., Braakman E., Peek A.M.L., van Noesel M.M. (2019). Peripheral stem cell apheresis is feasible post (131)iodine-metaiodobenzylguanidine-therapy in high-risk neuroblastoma, but results in delayed platelet reconstitution. Clin. Cancer Res..

[41] Kamran N., Li Y., Sierra M., Alghamri M.S., Kadiyala P., Appelman H.D., Edwards M., Lowenstein P.R., Castro M.G. (2018). Melanoma induced immunosuppression is mediated by hematopoietic dysregulation. Oncoimmunology.

[42] Guo J., Zhao Y., Fei C., Zhao S., Zheng Q., Su J., Wu D., Li X., Chang C. (2018). Dicer1 downregulation by multiple myeloma cells promotes the senescence and tumor-supporting capacity and decreases the differentiation potential of mesenchymal stem cells. Cell. Death Dis..

[43] Desbourdes L., Javary J., Charbonnier T., Ishac N., Bourgeais J., Iltis A., Chomel J.C., Turhan A., Guilloton F., Tarte K. (2017). Alteration analysis of bone marrow mesenchymal stromal cells from de novo acute myeloid leukemia patients at diagnosis. Stem. Cells Dev..

[44] Cordeiro-Spinetti E., de Mello W., Trindade L.S., Taub D.D., Taichman R.S., Balduino A. (2014). Human bone marrow mesenchymal progenitors: Perspectives on an optimized in vitro manipulation. Front. Cell. Dev. Biol..

[45] Roodman G.D. (2004). Mechanisms of bone metastasis. Discov. Med..

[46] Conforti A., Biagini S., Del Bufalo F., Sirleto P., Angioni A., Starc N., Li Pira G., Moretta F., Proia A., Contoli B. (2013). Biological, functional and genetic characterization of bone marrow-derived mesenchymal stromal cells from pediatric patients affected by acute lymphoblastic leukemia. PLoS ONE.

[47] Battula V.L., Le P.M., Sun J.C., Nguyen K., Yuan B., Zhou X., Sonnylal S., McQueen T., Ruvolo V., Michel K.A. (2017). Aml-induced osteogenic differentiation in mesenchymal stromal cells supports leukemia growth. JCI Insight.

[48] Hall C.L., Bafico A., Dai J., Aaronson S.A., Keller E.T. (2005). Prostate cancer cells promote osteoblastic bone metastases through wnts. Cancer Res..

[49] Wessely A., Waltera A., Reichert T.E., Stockl S., Grassel S., Bauer R.J. (2019). Induction of alp and mmp9 activity facilitates invasive behavior in heterogeneous human bmsc and hnscc 3d spheroids. Faseb. J..

[50] HaDuong J.H., Blavier L., Baniwal S.K., Frenkel B., Malvar J., Punj V., Sposto R., DeClerck Y.A. (2015). Interaction between bone marrow stromal cells and neuroblastoma cells leads to a vegfa-mediated osteoblastogenesis. Int. J. Cancer.

[51] Lu Y., Zhang J., Dai J., Dehne L.A., Mizokami A., Yao Z., Keller E.T. (2004). Osteoblasts induce prostate cancer proliferation and psa expression through interleukin-6-mediated activation of the androgen receptor. Clin. Exp. Metastasis.

[52] Larsen K.H., Frederiksen C.M., Burns J.S., Abdallah B.M., Kassem M. (2010). Identifying a molecular phenotype for bone marrow stromal cells with in vivo bone-forming capacity. J. Bone Min. Res..

[53] Correa D., Somoza R.A., Lin P., Schiemann W.P., Caplan A.I. (2016). Mesenchymal stem cells regulate melanoma cancer cells extravasation to bone and liver at their perivascular niche. Int. J. Cancer.

[54] Kucerova L., Zmajkovic J., Toro L., Skolekova S., Demkova L., Matuskova M. (2015). Tumor-driven molecular changes in human mesenchymal stromal cells. Cancer Microenviron..

[55] Schiano C., Grimaldi V., Casamassimi A., Infante T., Esposito A., Giovane A., Napoli C. (2012). Different expression of cd146 in human normal and osteosarcoma cell lines. Med. Oncol..

[56] Piskareva O., Harvey H., Nolan J., Conlon R., Alcock L., Buckley P., Dowling P., Henry M., O’Sullivan F., Bray I. (2015). The development of cisplatin resistance in neuroblastoma is accompanied by epithelial to mesenchymal transition in vitro. Cancer Lett..

[57] Burchill S.A., Beiske K., Shimada H., Ambros P.F., Seeger R., Tytgat G.A., Brock P.R., Haber M., Park J.R., Berthold F. (2017). Recommendations for the standardization of bone marrow disease assessment and reporting in children with neuroblastoma on behalf of the international neuroblastoma response criteria bone marrow working group. Cancer.

[58] Cheung N.K., Ostrovnaya I., Kuk D., Cheung I.Y. (2015). Bone marrow minimal residual disease was an early response marker and a consistent independent predictor of survival after anti-gd2 immunotherapy. J. Clin. Oncol..

[59] Fried W., Chamberlin W., Kedo A., Barone J. (1976). Effects of radiation on hematopoietic stroma. Exp. Hematol..

[60] Domenech J., Roingeard F., Herault O., Truglio D., Desbois I., Colombat P., Binet C. (1998). Changes in the functional capacity of marrow stromal cells after autologous bone marrow transplantation. Leuk. Lymphoma.

[61] Somaiah C., Kumar A., Sharma R., Sharma A., Anand T., Bhattacharyya J., Das D., Deka Talukdar S., Jaganathan B.G. (2018). Mesenchymal stem cells show functional defect and decreased anti-cancer effect after exposure to chemotherapeutic drugs. J. Biomed. Sci..

[62] Prata Kde L., Orellana M.D., De Santis G.C., Kashima S., Fontes A.M., Carrara Rde C., Palma P.V., Neder L., Covas D.T. (2010). Effects of high-dose chemotherapy on bone marrow multipotent mesenchymal stromal cells isolated from lymphoma patients. Exp. Hematol..

[63] Maijenburg M.W., Noort W.A., Kleijer M., Kompier C.J., Weijer K., van Buul J.D., van der Schoot C.E., Voermans C. (2010). Cell cycle and tissue of origin contribute to the migratory behaviour of human fetal and adult mesenchymal stromal cells. Br. J. Haematol..

[64] Stutterheim J., Gerritsen A., Zappeij-Kannegieter L., Yalcin B., Dee R., van Noesel M.M., Berthold F., Versteeg R., Caron H.N., van der Schoot C.E. (2009). Detecting minimal residual disease in neuroblastoma: The superiority of a panel of real-time quantitative pcr markers. Clin. Chem..

[65] Bate-Eya L.T., Ebus M.E., Koster J., den Hartog I.J., Zwijnenburg D.A., Schild L., van der Ploeg I., Dolman M.E., Caron H.N., Versteeg R. (2014). Newly-derived neuroblastoma cell lines propagated in serum-free media recapitulate the genotype and phenotype of primary neuroblastoma tumours. Eur. J. Cancer.

[66] Van Nes J., Chan A., van Groningen T., van Sluis P., Koster J., Versteeg R. (2013). A notch3 transcriptional module induces cell motility in neuroblastoma. Clin. Cancer Res..

