# Supplementary figures and images for: The Metastatic Bone Marrow Niche in Neuroblastoma: Altered Phenotype and Function of Mesenchymal Stromal Cells
Source: Cancers (Basel). 2020 Nov 2;12(11):3231. doi: 10.3390/cancers12113231 (PMC7692745; doi:10.3390/cancers12113231)

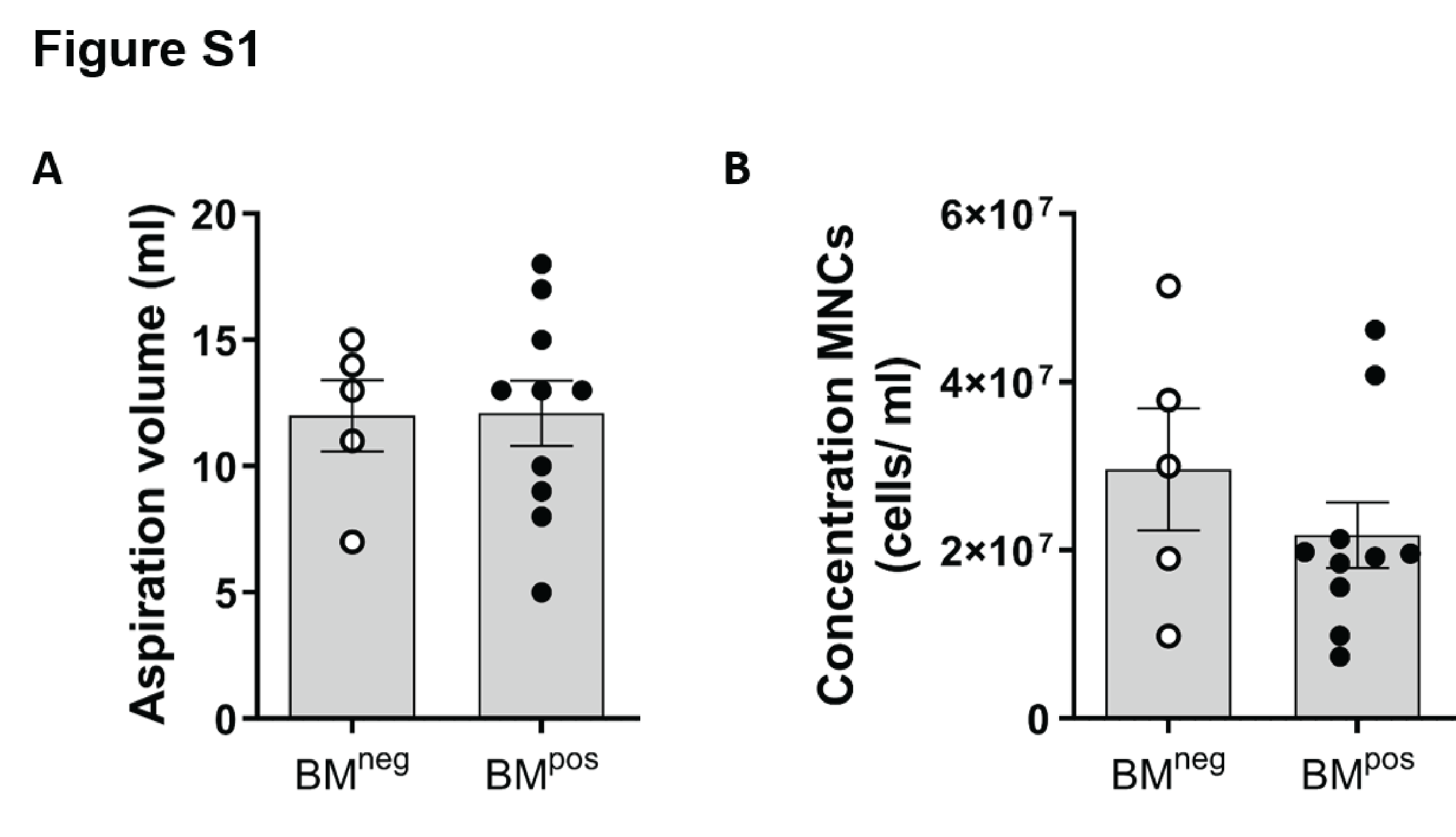

Supplement: Supplementary file 1 [file cancers-12-03231-s001.zip › Figure S1 300dpi.tif]

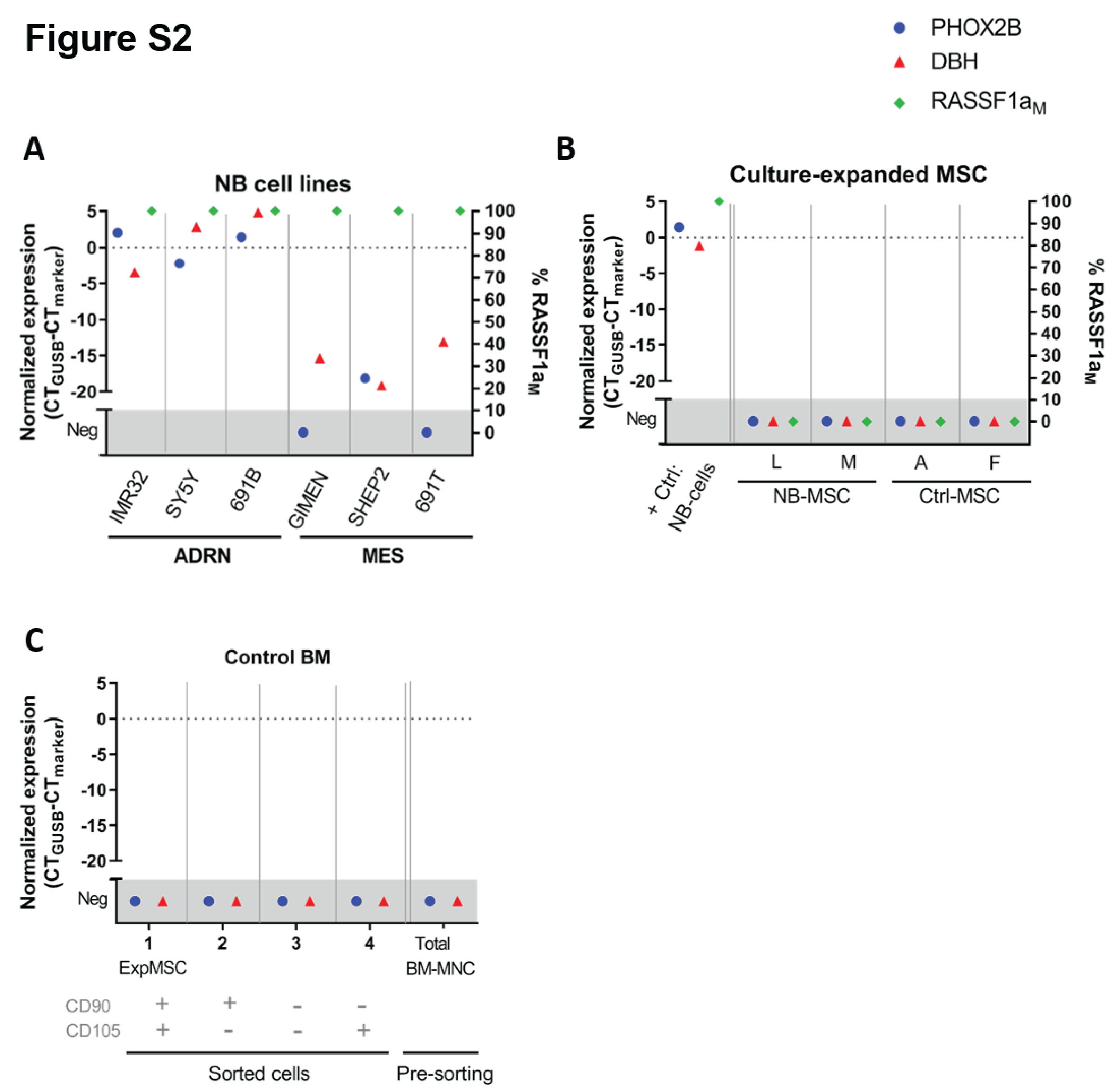

Supplement: Supplementary file 1 [file cancers-12-03231-s001.zip › Figure S2 300dpi.tif]

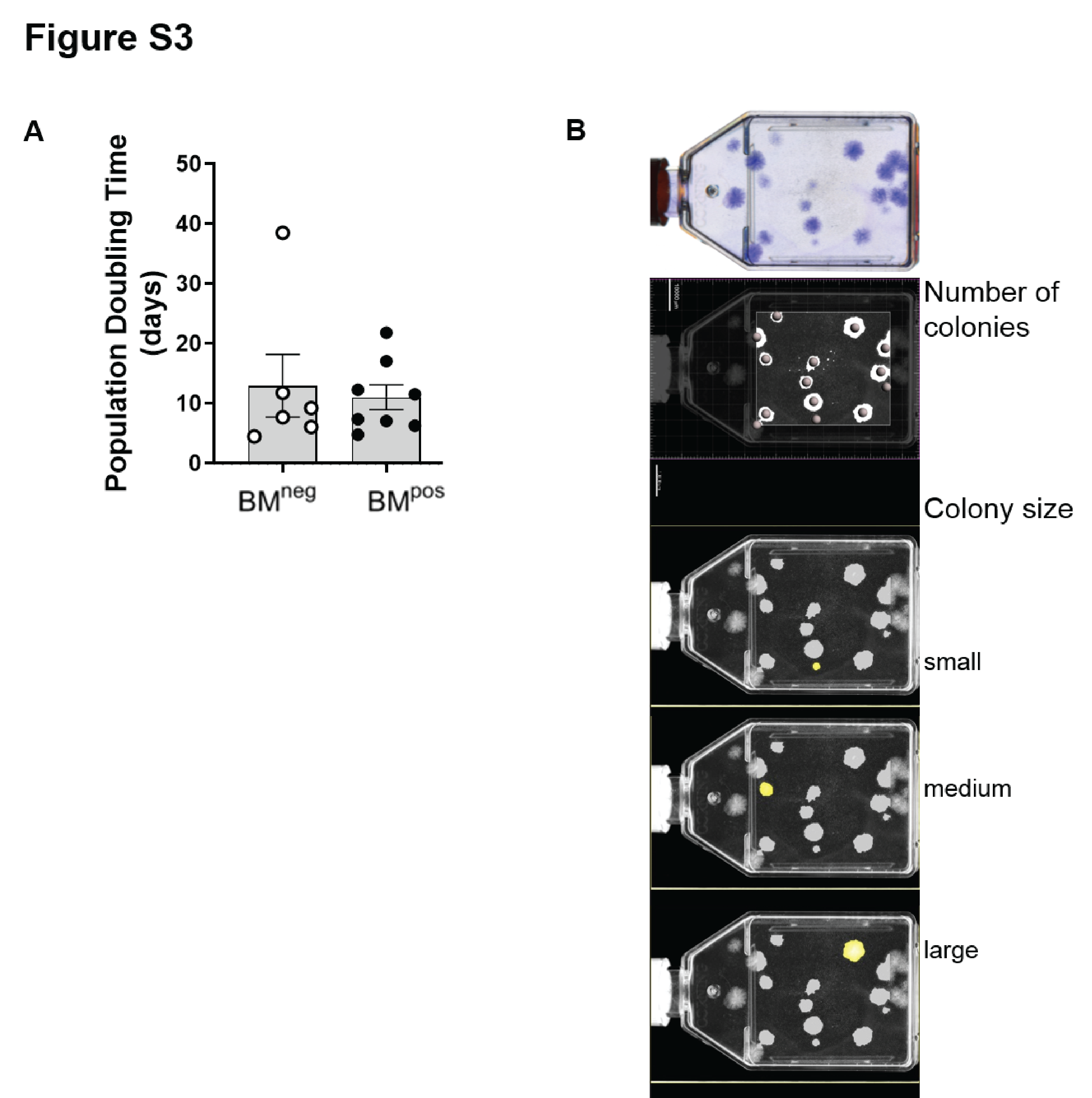

Supplement: Supplementary file 1 [file cancers-12-03231-s001.zip › Figure S3 300dpi.tif]

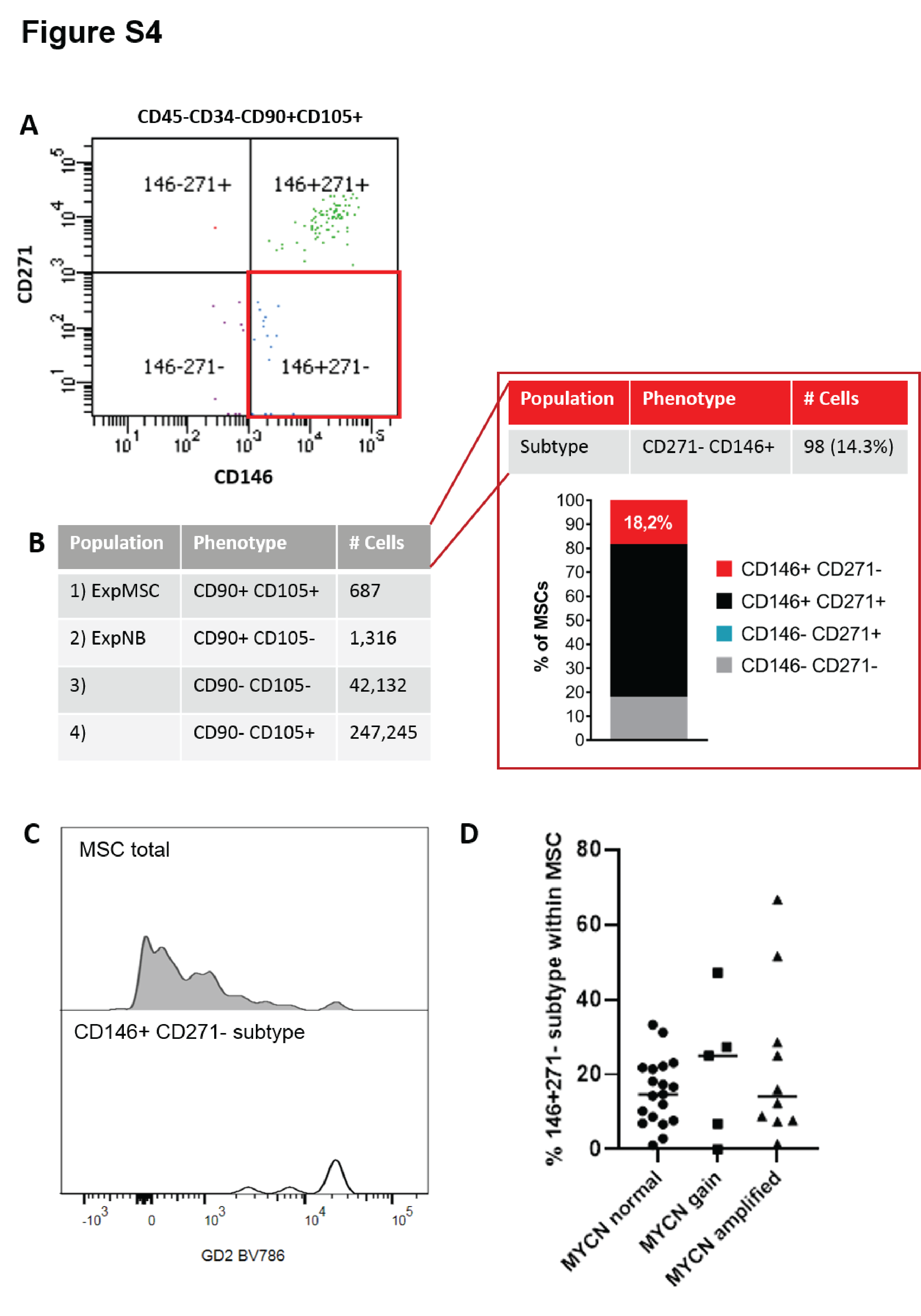

Supplement: Supplementary file 1 [file cancers-12-03231-s001.zip › Figure S4 300dpi.tif]

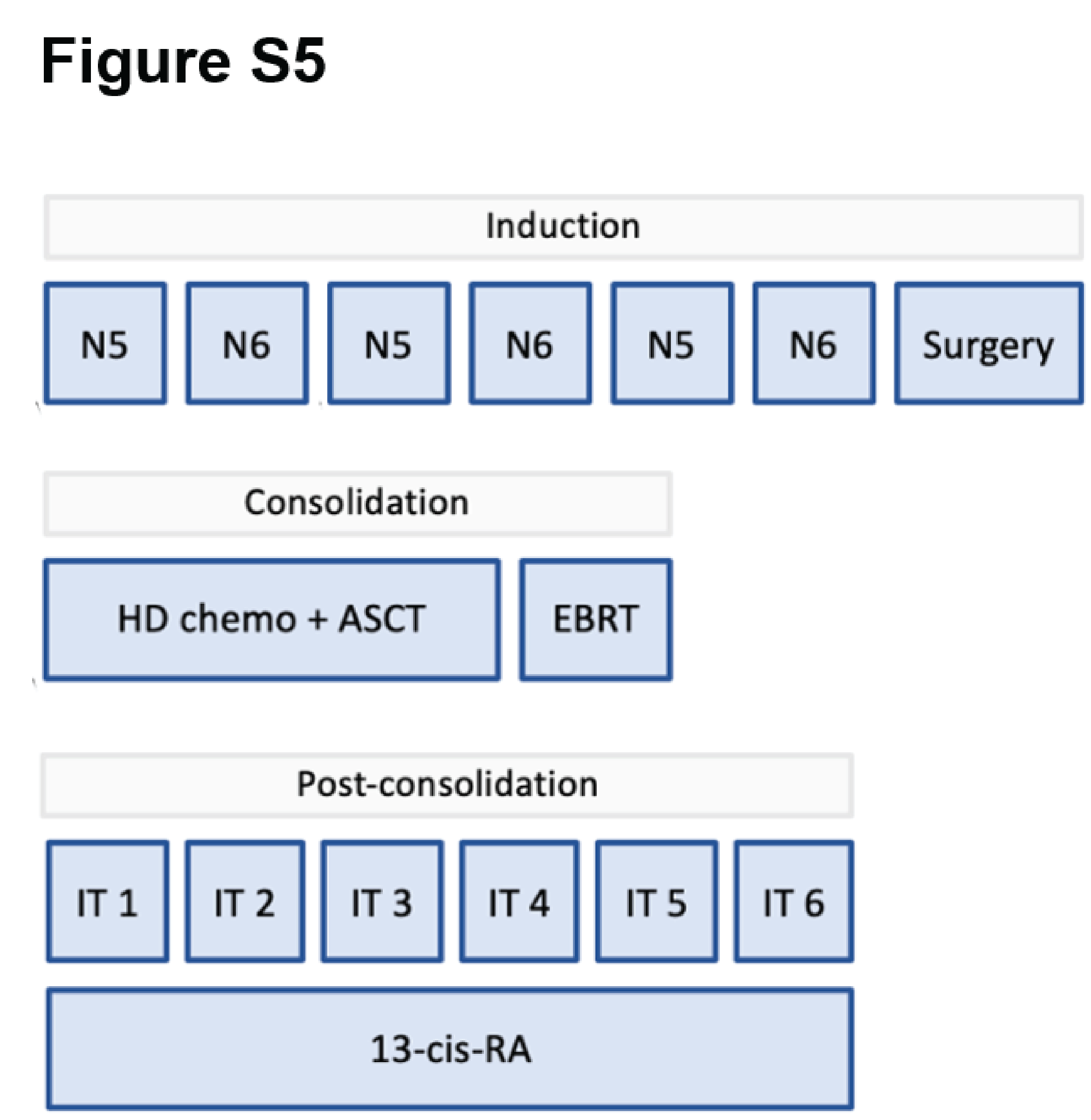

Supplement: Supplementary file 1 [file cancers-12-03231-s001.zip › Figure S5 300dpi.tif]

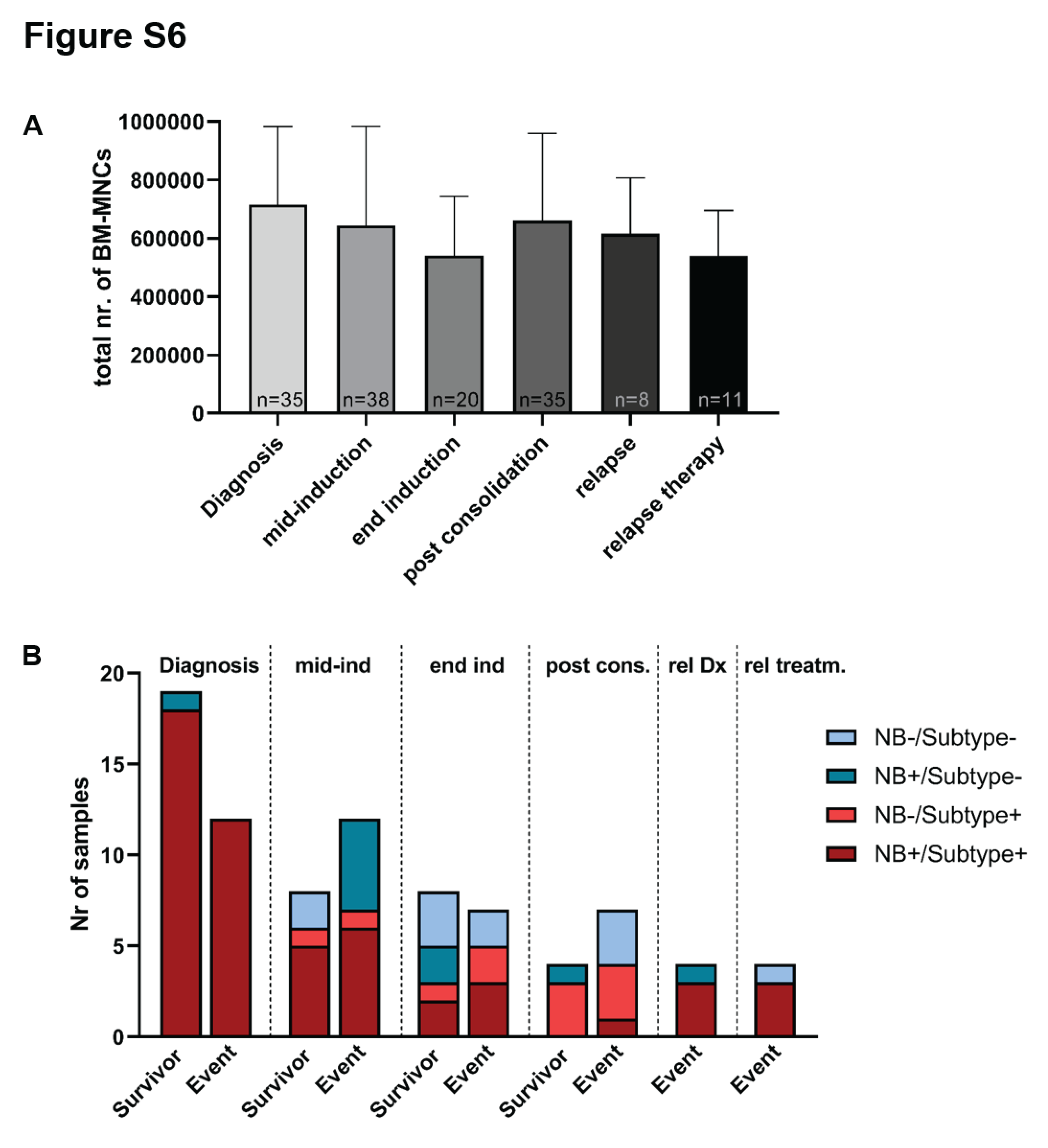

Supplement: Supplementary file 1 [file cancers-12-03231-s001.zip › Figure S6 300dpi.tif]

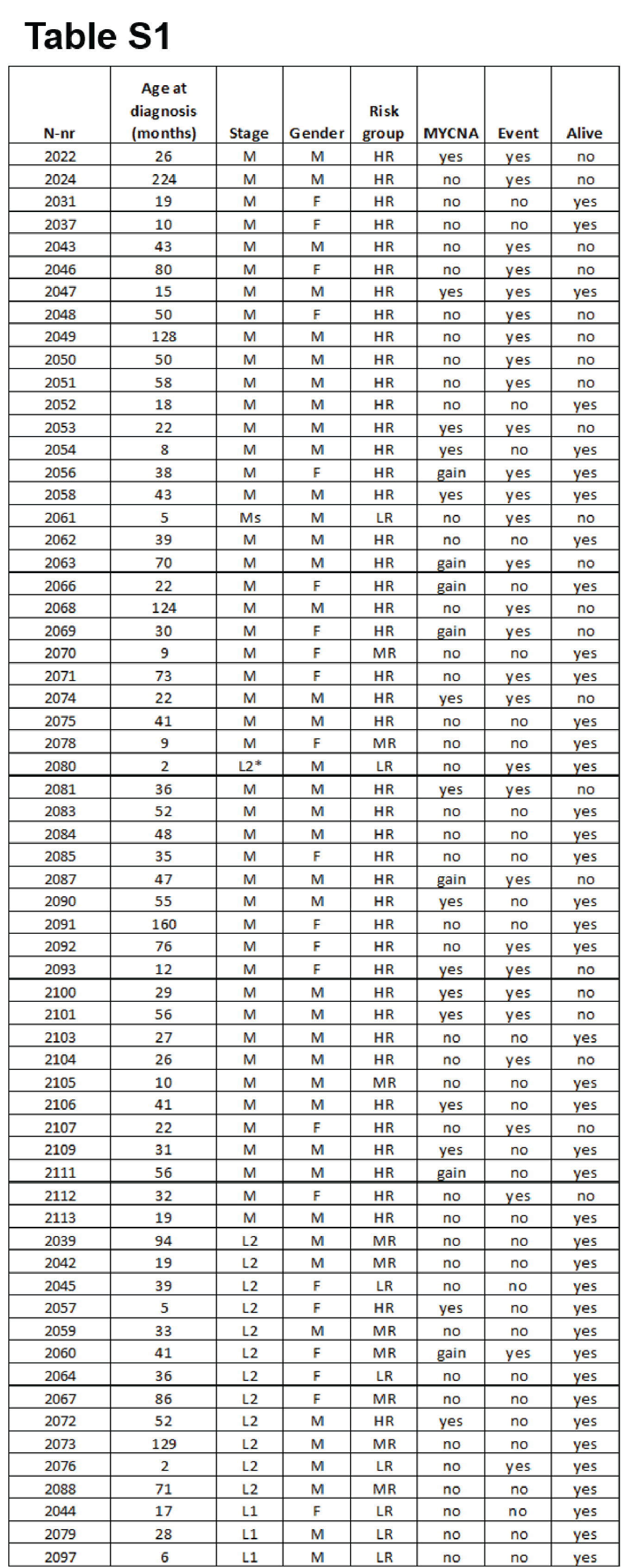

Supplement: Supplementary file 1 [file cancers-12-03231-s001.zip › Table S1 300dpi.tif]

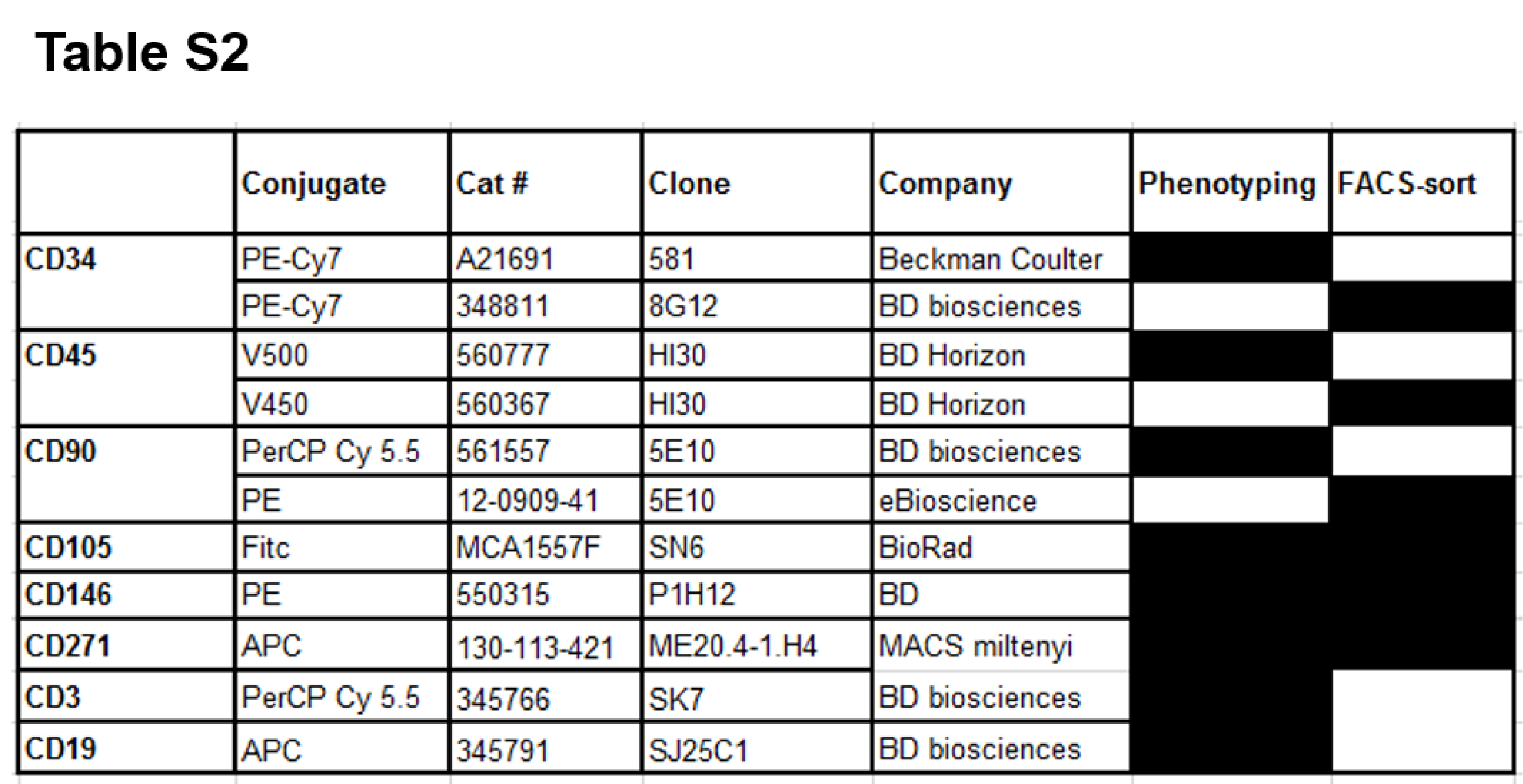

Supplement: Supplementary file 1 [file cancers-12-03231-s001.zip › Table S2 300dpi.tif]

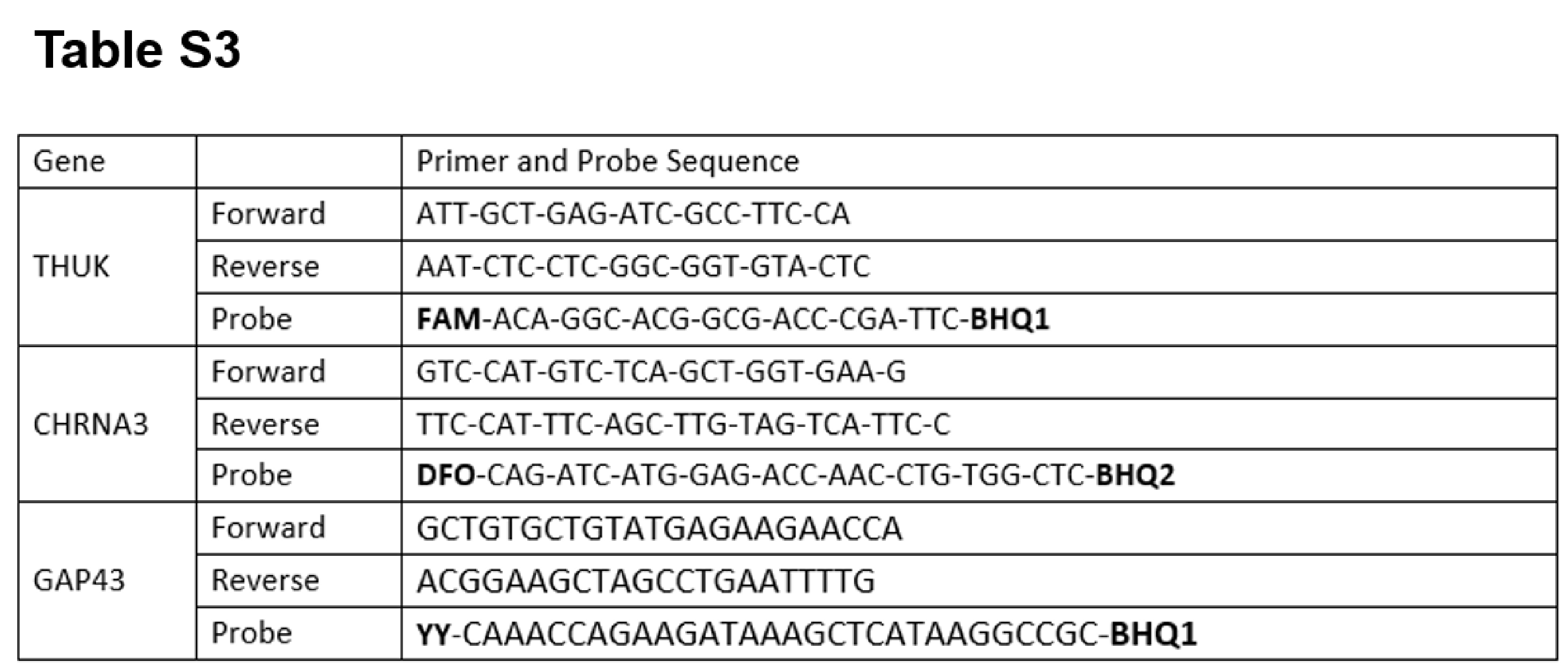

Supplement: Supplementary file 1 [file cancers-12-03231-s001.zip › Table S3 300dpi.tif]
